# Nitrate-dependent antimony oxidase in an uncultured *Symbiobacteriaceae* member

**DOI:** 10.1093/ismejo/wrae204

**Published:** 2024-10-16

**Authors:** Liying Wang, Zhipeng Yin, Wei Yan, Jialong Hao, Fei Tian, Jianbo Shi

**Affiliations:** State Key Laboratory of Environmental Chemistry and Ecotoxicology, Research Center for Eco-Environmental Sciences, Chinese Academy of Sciences, Beijing, China; State Key Laboratory of Environmental Chemistry and Ecotoxicology, Research Center for Eco-Environmental Sciences, Chinese Academy of Sciences, Beijing, China; State Key Laboratory of Environmental Chemistry and Ecotoxicology, Research Center for Eco-Environmental Sciences, Chinese Academy of Sciences, Beijing, China; Key Laboratory of Earth and Planetary Physics, Institute of Geology and Geophysics, Chinese Academy of Sciences, Beijing, China; CAS Engineering Laboratory for Deep Resources Equipment and Technology, Institute of Geology and Geophysics, Chinese Academy of Sciences, Beijing, China; State Key Laboratory of Environmental Chemistry and Ecotoxicology, Research Center for Eco-Environmental Sciences, Chinese Academy of Sciences, Beijing, China; School of Environment, Hangzhou Institute for Advanced Study, University of Chinese Academy of Sciences, Hangzhou, China; MOE Key Laboratory of Groundwater Quality and Health, School of Environmental Studies, China University of Geosciences, Wuhan, China

**Keywords:** nitrate-dependent Sb(III) oxidation, *Symbiobacteriaceae*, antimony oxidase

## Abstract

Autotrophic antimony (Sb) oxidation coupled to nitrate reduction plays an important role in the transformation and detoxification of Sb. However, the specific oxidase involved in this process has yet to be identified. Herein, we enriched the microbiota capable of nitrate-dependent Sb(III) oxidation and identified a new Sb(III) oxidase in an uncultured member of *Symbiobacteriaceae*. Incubation experiments demonstrated that nitrate-dependent Sb(III) oxidation occurred in the microcosm supplemented with Sb(III) and nitrate. Both the 16S rRNA gene and metagenomic analyses indicated that a species within *Symbiobacteriaceae* played a crucial role in this process. Furthermore, carbon-13 isotope labeling with carbon dioxide–fixing *Rhodopseudomonas palustris* in combination with nanoscale secondary ion mass spectrometry revealed that a newly characterized oxidase from the dimethylsulfoxide reductase family, designated as NaoABC, was responsible for autotrophic Sb(III) oxidation coupled with nitrate reduction. The NaoABC complex functions in conjunction with the nitrate reductase NarGHI, forming a redox loop that transfers electrons from Sb(III) to nitrate, thereby generating the energy necessary for autotrophic growth. This research offers new insights into the understanding of how microbes link Sb and nitrogen biogeochemical cycles in the environment.

## Introduction

Antimony (Sb) is a toxic metalloid and listed as a priority pollutant by both the United States Environmental Protection Agency and the European Union [1]. Sb contamination is ubiquitous worldwide due to the ever-increasing industrial activities [2]. Sb toxicity mainly depends on its chemical speciation [3]. In particular, Sb(III) shows greater toxicity and bioavailability, resulting in a higher uptake and accumulation in the crops than Sb(V) [4, 5]. Therefore, Sb(III) oxidation is of great importance in diminishing the risk of Sb toxicity to health.

Sb(III) oxidation is driven by microorganisms [6, 7]. Diverse heterotrophic microorganisms are capable of oxidizing Sb(III) to Sb(V) by oxidoreductases AioAB [8] and AnoA [9]. However, in anoxic conditions, some autotrophic organisms could couple Sb(III) oxidation and nitrate reduction, such as *Ensifer* [10] and *Sinorhizobium* [11]. Recently, three new members, *Azoarcus*, *Azospira*, and *Chelativorans*, were identified as the contributors for nitrate-dependent Sb(III) oxidation in a Sb-contaminated paddy soil by metagenomic binning analysis. Additionally, *aioA* was suggested to be involved in nitrate-dependent Sb oxidation [2]. However, some *aioA*-hosting bacteria, such as *Hydrogenophaga taeniospiralis* IDSBO-1, were unable to perform nitrate-dependent Sb(III) oxidation [12], implying the existence of as yet unknown enzymatic pathways. The lack of such a mechanistic basis has hindered previous attempts to gain molecular insights into nitrate-dependent Sb(III) oxidation which contributes to the fate and mobility of Sb.

The dimethylsulfoxide reductase (DMSOR) is an important enzyme catalyzing the oxidation/reduction transformation of many elements, such as carbon (C), nitrogen (N), sulfur (S), and arsenic (As) [13], because of its unique composition and structure. In general, DMSOR is a heterotrimeric complex comprising a Mo/W-bisPGD-harboring subunit for catalytic reactions, an electron transfer subunit of [Fe-S] clusters, and an electron entry/exit subunit binding to the cell membrane which links the complex to the electron transport chain [14]. However, many new and distinct clades of the DMSOR family in prokaryotic genomes remain uncharacterized, indicating that the importance of DMSOR in microbial metabolisms may be underestimated. With regard to the element Sb, both Sb(III) oxidase (AioAB [8]) and Sb(V) respiratory reductase (ArrAB [15], AnrA [14]) belong to the DMSOR family, suggesting that the enzyme responsible for nitrate-dependent Sb(III) oxidation may be a member of the DMSOR family.

The objective of this study was to identify the nitrate-dependent Sb(III) oxidizing microbes and oxidase. Here, we enriched the nitrate-dependent Sb(III) oxidizing microbiota from Sb-contaminated soil by an anoxic microcosm experiment. An uncultivated *Symbiobacteriaceae* species was identified as a crucial functional organism by the 16S rRNA gene and metagenomic analyses. Moreover, a subclass of the DMSOR family (designated as NaoABC) was characterized as a new oxidase for autotrophic Sb(III) oxidation by ^13^C labeling with CO_2_-fixing *Rhodopseudomonas palustris* in combination with nanoscale secondary ion mass spectrometry technology. These findings contribute an essential step in our comprehension of the enzymatic pathway for nitrate-dependent Sb(III) oxidation that controls the fate of Sb in the anoxic environments.

## Materials and methods

### Soil collection and characterization

Soil samples were collected at a depth of 40–60 cm below the soil surface from five sampling points in the vicinity of an active Sb mine in Lengshuijiang, China (27°47′29″ N; 111°30′14″ E). Sampled soils were quickly enclosed in sterile plastic bags (Bkmanlab, China), thoroughly mixed, transported at 4°C, and stored at −80°C before the characterization analysis and the anoxic incubation experiment. The main components of the samples are listed in Table S1.

### Anoxic incubation experiment

To assess the capability of nitrate-dependent Sb(III), an anoxic incubation experiment was carried out. Approximately 2 g of soil was incubated with 100 mL mineral salts medium (MSM). The specific components of MSM are detailed in the Supplementary Information (SI). Six treatments (*n* = 3 each) were set up: (i) soil: soil incubated with MSM; (ii) soil + Sb(III): soil treatment amended with 1 mM antimony potassium tartrate [Sb(III)]; (iii) soil + ^15^NO_3_^−^: soil treatment amended with 10 mM ^15^KNO_3_ [NO_3_^−^]; (iv) soil + Sb(III) + ^15^NO_3_^−^: soil treatment amended with 1 mM Sb(III) and 10 mM ^15^NO_3_^−^; (v) negative control soil: the sterilized soil incubated with MSM amended with 1 mM Sb(III) and 10 mM ^15^NO_3_^−^; (vi) soil + Sb(III) + ^15^NO_3_^−^ + azide: soil + Sb(III) + ^15^NO_3_^−^ treatment amended with 10 mM azide. All the microcosms were incubated at 30°C in a 100% N_2_ atmosphere in the dark without shaking for 8 days. Cultures were periodically sampled and analyzed for the concentrations of Sb(V), Sb(III), NO_3_^−^, NO_2_^−^, Fe(II), and absorbed Fe(III) [labile Fe(III)] [16]. At the end of the incubation, ^15^N_2_O and ^15^N_2_ in the headspace air, and ^15^NH_4_^+^ from dissimilatory nitrate reduction to ammonium (DNRA) [17] were sampled for measurement. The precipitates were collected and washed three times with MSM medium for the experiment on Sb(III) oxidation by labile Fe(III). All operations were performed under a 100% N_2_ atmosphere.

To assess the abiotic Sb(III) oxidation, separate incubation experiments in 100 mL MSM were conducted as follows: 1 mM Sb(III) was mixed with 1 mM Fe(III), 1 mM Sb(III) was mixed with 10 mM nitrite, and 1 mM Sb(III) was combined with the precipitates from the above soil microcosms. Additionally, 10 mM azide was added to the incubation containing the precipitates to prevent any microbial activity. All the incubations were performed at 30°C under a 100% N_2_ atmosphere in the dark without shaking for 8 days. Periodically, 1 mL of the suspension was sampled with a sterile needle to determine the concentrations of Sb(V) and Sb(III).

### Determination of Sb, Fe, and nitrogen species

The collected samples were filtered through a sterile 0.22-μm filter for the quantification of Sb, Fe, and nitrogen species. The concentrations of Sb(III) and Sb(V) were determined using HPLC coupled to atomic fluorescence spectrometry (AFS) [18] as described in SI. Fe(II) was quantified using the ferrozine colorimetric assay at 562 nm [19]. Labile Fe(III) was assayed as previously described [16]. For the detection of NO_2_^−^, sulfanilamide and *N*-(1-naphthyl)-ethylenediamine dihydrochloride were used to form a deep purple azo compound, which was detected at 540 nm [20]. NO_3_^−^ was measured by ion chromatography with conductivity detection. The analysis was performed on a Dionex ICS-2000 ion chromatograph configured with a Dionex AS-18 column and 32 mM KOH mobile phase flowing at 1.0 mL/min [21]. N_2_O was measured using a gas chromatograph (Agilent 7890 A; Agilent Inc., USA) by a micro-electron capture detector as previously described [22]. ^15^N_2_ was measured by isotope ratio mass spectrometry (MAT253 and Gasbench II; Thermofisher Scientific, Bremen, Germany) at the Key Laboratory of Tibetan Environment Changes and Land Surface Processes, Institute of Tibetan Plateau Research, Chinese Academy of Sciences [23]. DNRA ammonium was measured by complete conversion of ^15^NH_4_^+^ to ^30^N_2_ in accordance with the previously described methodology [23].

### 16S rRNA gene amplicon sequencing

To explore the putative bacteria capable of nitrate-dependent Sb(III) oxidation, the 16S rRNA gene sequencing and analysis of the microbial incubation treatments were carried out. Genomic DNA of microbes from the treatments on day 8 was extracted using a FastDNA Spin Kit (TIANGEN BIOTECH, Beijing) according to the manufacturer’s protocol. The V3 and V4 regions of the 16S rRNA genes were amplified using the extracted DNA as a template, and then subjected to high-throughput sequencing. Details of the PCR amplification, sequencing, and sequence analyses using QIIME2 are described in SI. Raw reads were deposited in the NCBI Sequence Read Archive (SRA) database (accession number: PRJNA862322).

### Metagenome sequencing and analysis

To identify the organisms and their associated genes that mediate nitrate-dependent Sb(III) oxidation in the microcosm of soil + Sb(III) + NO_3_^−^, metagenome sequencing and binning analyses were performed. DNA from the microcosm of soil + Sb(III) + NO_3_^−^ on day 8 was extracted and sequenced for the metagenome on an NovaSeq platform (Illumina, San Diego, USA) according to the standard protocol of Majorbio Bio-Pharm Technology Co. Ltd. (Shanghai, China). Raw reads (10 Gb) were trimmed and filtered using fastp [24]. The qualified reads were assembled into contigs using Megahit (v1.1.2) (*k* = 21–121, step = 10) [25]. Binning of metagenomic contigs was carried out using CONCOCT software [26]. Completeness/contamination calculation, identification of essential single-copy genes, and coverage distribution of the resulting genome bins were assessed using CheckM (v0.9.7) [27]. Only high-quality bins with completion >70% and contamination <5% were selected for further analysis. Taxonomy of the bins was classified using Taxator-tk [28]. Annotation of the bins was performed using KofamKOALA online (score > 60, e value = 1e−5) [29]. In addition, the phylogenetic tree of the bins was constructed based on conserved protein sequences using GTDB-Tk (v2.3.0) [30]. The metagenomic reads were deposited in the NCBI SRA under accession number PRJNA862653.

### Nitrate-dependent Sb(III) oxidation in recombinant *R. palustris*

To elucidate the role of NaoABC in the nitrate-dependent Sb(III) oxidation process, and to confirm that this process can generate energy for microbial proliferation, a heterologous expression experiment was carried out using *R. palustris*, which is capable of fixing carbon dioxide. For the construction of the recombinant *R. palustris*, the genomic DNA of the soil + Sb(III) + NO_3_^−^ microcosm on day 8 was extracted and used as a template for the amplification of the two *nao* clusters. The full length of *nao1* (5.5 kb), *nao2* (6.7 kb) containing their own promoters and the contiguous genes of naoABC were amplified, respectively. The primers used are listed in Table S2. The PCR products were cloned into the plasmid pCE2TA/Blunt-Zero vector using a TA/Blunt-Zero cloning kit (Vazyme, Nanjing) according to the manufacturer’s protocol, resulting in the recombinant plasmids pCE2TA-*nao1* and pCE2TA-*nao2*. The two recombinant plasmids were then transformed into the carbon-fixing strain *R. palustris* possessing the *narGHI* cluster (SI), acquiring the recombinant *R. palustris-*pCE2TA*-nao1* and *R. palustris-*pCE2TA*-nao2*, respectively. *Rhodopseudomonas palustris-*pCE2TA without *nao* genes was used as a control.

For nitrate-dependent Sb(III) oxidation in *R. palustris*, the engineered *R. palustris* strains were cultured in SLM medium for 24 h, harvested, washed three times, and resuspended in MSM medium, respectively. The subsequent incubation experiment was performed by adding 1 mL of the cell suspension to 20 mL of fresh MSM medium (10 mM NaH^13^CO_3_) supplemented with 0.1 mM Sb(III) and 10 mM NO_3_^−^ at 30°C under 100% N_2_ atmosphere in the dark without shaking for 8 days. To enhance the capacity of nitrate-dependent Sb(III) oxidation, 0.2 mM IPTG was added to induce overexpression of the *nao* genes. Cultures were sampled periodically and analyzed for cell number as described in SI. At the end of incubation, 1 mL of the suspension was sampled with a sterile needle to determine the concentrations of Sb(V) and Sb(III) by AFS [18]. In addition, the biomass was collected and washed three times with phosphate-buffered saline, followed by ^13^C incorporation analysis by the isotope ratio mass spectrometry [31] and visualization analysis through the nanoscale secondary ion mass spectrometry (nano-SIMS) [32].

### Phylogenetic methods

To analyze the evolution of NaoABC, sequences were aligned using CLUSTAL W [33] and maximum-likelihood phylogenetic trees were constructed using RAxML (v8.2.12) [34]. The significance of branch points was estimated from 1000 bootstrap replications [35].

### Statistical analysis

Correlation analysis was performed using IBM SPSS Statistics, version 20.0. Kruskal–Wallis sum-rank [36] was used to determine the possible significant differences among the four microcosms of soil, soil + Sb(III), soil + NO_3_^−^, and soil + Sb(III) + NO_3_^−^. Significance was considered when *P* <.05.

## Results

### Microbial Sb(III) oxidation coupled to NO_3_^−^ reduction

The soil sample containing 0.07 mmol/g Sb(V) (Table S1) was incubated in MSM amended with Sb(III) and nitrate under anoxic conditions for 8 days. Gas bubbles were clearly observed in the nitrate-amended soil samples, but not in the other soil samples (Fig. S1), indicating the microbial denitrification [37]. More gas bubbles were observed when both Sb(III) and nitrate were added (Fig. S1D), suggesting that Sb(III) enhanced the microbial denitrification. This is probably due to the coupling of Sb(III) oxidation with nitrate reduction.

**Figure 1:**
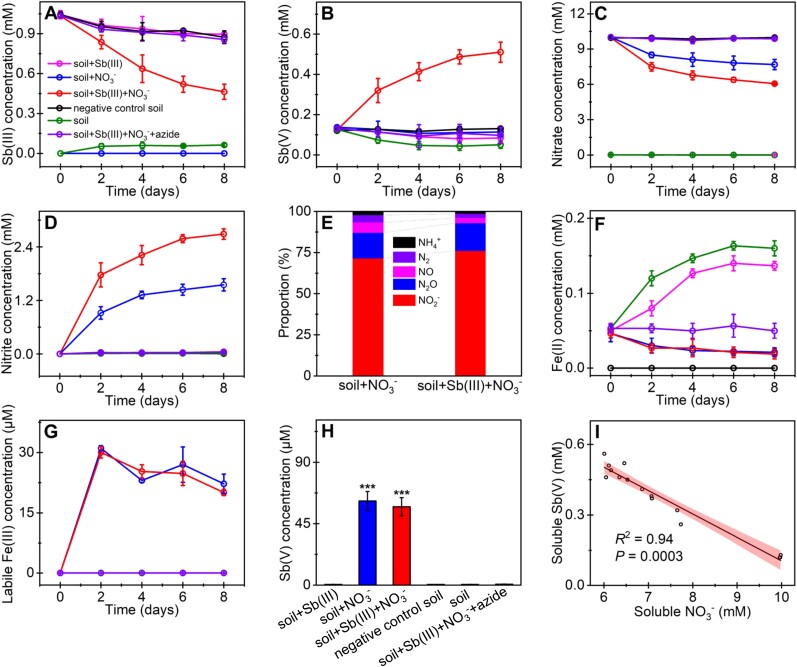
The detection of microbial Sb(III) oxidation coupled to NO_3_^−^ reduction. Time-dependent concentration of dissolved Sb(III) (**A**), Sb(V) (**B**), NO_3_^−^ (**C**), and NO_2_^−^ (**D**) in the microcosm of soil + Sb(III), soil + NO_3_^−^, soil + Sb(III) + NO_3_^−^, negative control soil, soil + Sb(III) + NO_3_^−^ + azide, and soil. The percentage of N products (**E**), NO was calculated by deducing those of NO_2_^−^, N_2_O, N_2_, and NH_4_^+^. Time-dependent concentration of dissolved Fe(II) (**F**), labile Fe(III) (**G**), and the amount of abiotic Sb(III) oxidation by labile Fe(III) as measured by adding 1 mM Sb(III) to the precipitates obtained from respective soil microcosm (**H**). Correlation analysis between soluble NO_3_^−^ and Sb(V) in soil + Sb(III) + NO_3_^−^ incubation (**I**). Error bars represent the standard deviations of means of triplicates.

To investigate the occurrence of coupling of Sb(III) oxidation with nitrate reduction, dissolved Sb(III/V), nitrate, and nitrite during the incubation were measured. No discernible Sb(III) oxidation was observed in the negative control soil, soil + Sb(III) + NO_3_^−^ + azide, soil + Sb(III), soil + NO_3_^−^, and soil microcosms. An appreciable Sb(III) oxidation was only observed with Sb(III) and nitrate amendment. Specifically, Sb(III) was reduced from 1.03 ± 0.01 mM at the beginning of the incubation to 0.46 ± 0.06 mM on day 8, and concurrently Sb(V) was increased to 0.51 ± 0.05 mM (Fig. 1A and B). Concurrently, a decrease in nitrate from 9.97 ± 0.01 to 6.06 ± 0.05 mM and an increase in nitrite up to 2.69 ± 0.12 mM were observed (Fig. 1C and D). These findings indicate that Sb(III) can serve as an electron donor for nitrate reduction, aligning with the results of a previous study [38].

The amendment of nitrate alone resulted in almost no change in Sb speciation, but a notable increase in nitrite up to 1.60 ± 0.14 mM (blue line in Fig. 1D), suggesting the potential involvement of other electron donors in nitrate reduction, including natural organic matter (Table S1) and microbial reduced Fe(II) (Fig. 1F). The reduced nitrite was subsequently reduced to NO, N_2_O, and N_2_ by denitrifying microbes. The final product of denitrification, ^15^N_2_, represented ~6% and 3% of the NO_3_^−^-reduced products in soil + NO_3_^−^ and soil + Sb(III) + NO_3_^−^, respectively (Fig. 1E). In addition to the denitrification pathway, nitrate/nitrite can be reduced by DNRA [17]. The presence of ^15^N labeled ammonium [2% in soil + NO_3_^−^ and 1% in soil + Sb(III) + NO_3_^−^] was minimal (Fig. 1E), indicating that DNRA activity was constrained or that any NH_4_^+^ produced was consumed or assimilated during the incubation period. Furthermore, the presence of N_2_O was observed in notable quantities, at ~16% for the soil + NO_3_^−^ treatment and 17% for the soil + Sb(III) + NO_3_^−^ treatment (Fig. 1E). This finding suggests that the chemical reaction of nitrite and Fe(II) (referred to as chemodenitrification [39]) was an obvious process in the microcosms with nitrate supplementation, consistent with the results of a previous study [40].

Microbial produced Fe(III) may contribute to abiotic Sb(III) oxidation [11], which is probably due to the adsorption of Fe(III) on the precipitates forming active labile Fe(III) [41]. This study revealed the occurrence of microbial Fe reduction in the microcosms of soil and soil + Sb(III) (Fig. 1F). In the presence of nitrate, the reduced Fe(II) decreased obviously (Fig. 1F), which is probably due to the nitrate-dependent Fe(II) oxidation [42]. The produced Fe(III) was adsorbed on the precipitates, forming active labile Fe(III) [about 31.07 for the soil + NO_3_^−^ and 30.03 μM for the soil + Sb(III) + NO_3_^−^ microcosm] (Fig. 1G). About 57.45 μM Sb(III) was oxidized abiotically by labile Fe(III) in the microcosm of soil + Sb(III) + NO_3_^−^, representing for 10% of the total Sb(III) oxidation (Fig. 1H). Consistent with the previous study [11], the intermediate products, nitrite and soluble Fe(III), are unable to oxidize Sb(III) (Fig. S2). Although many of the aforementioned reactions are involved in the microcosms, a significant negative correlation is observed between Sb(III) oxidation and nitrate reduction (*P* = .0003, Fig. 1I). These results suggest the occurrence of microbial nitrate-dependent Sb(III) oxidation in the microcosm supplemented with Sb(III) and nitrate. This process was further validated in the passage cultures. After eliminating the potential influence of labile Fe(III) in the third generation, nitrate-dependent Sb(III) oxidation was observed to persist (Fig. S3), despite a decreased Sb(III) oxidation intensity resulting from the low biomass observed during the cultivation period.

The process of nitrate-dependent Sb(III) oxidation can be described as follows:


$$ \mathrm{Sb}{\left(\mathrm{OH}\right)}_3+\kern0.33em {{\mathrm{NO}}_3}^{-}+\kern0.33em 2{\mathrm{H}}_2\mathrm{O}\to \mathrm{Sb}{{\left(\mathrm{OH}\right)}_6}^{-}+\kern0.33em {{\mathrm{NO}}_2}^{-}+\kern0.33em {\mathrm{H}}^{+} $$

The standard Gibbs free energy (Δ*G*°) of the reaction is −135 kJ/mol. The actual Gibbs free energy (Δ*G*) is calculated to be −147 kJ/mol (Table S3), which is corrected for the effects of concentration, speciation, and activity [43]. This result implies that the nitrate-dependent Sb(III) oxidation is thermodynamically favorable under the incubation conditions where Sb(III) is used as an electron donor for microbial nitrate reduction under anoxic conditions.

### Putative bacteria responsible for nitrate-dependent Sb(III) oxidation

High-throughput sequencing and analysis based on the 16S rRNA gene were carried out. The rarefaction curves (Fig. S4) with high coverage suggest that the sequencing depth was sufficient. The total number of identified taxa in the microcosms was relatively low. This is presumably attributable to contamination by Sb [44, 45], as well as the storage of the samples at −80°C [46]. The addition of Sb(III) and nitrate resulted in a notable reduction in microbial diversity, as evidenced by the significant difference in the Sobs and Shannon indexes (*P* < .001, Fig. S5). In contrast, no obvious difference was observed between the soil and the Sb(III) amendment (*P* = .08). Collectively, the results suggest that the amendment of Sb(III) and nitrate obviously altered the community structure, enriching the adapted microorganisms, which may include the functional bacteria responsible for coupled Sb(III) oxidation and NO_3_^−^ reduction.

**Figure 2:**
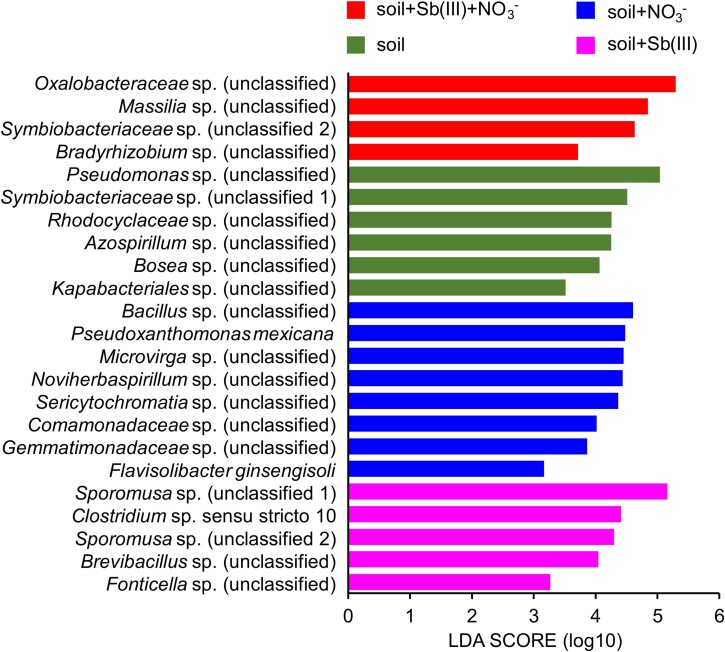
Identification of functional microbes for nitrate-dependent Sb(III) oxidation by LEfSe analysis. Comparative analysis of the differential abundance microbes at the species level among microcosms of soil, soil + NO_3_^−^, soil + Sb(III), and soil + Sb(III) + NO_3_^−^. The term “unclassified” refers to microbial strains that have not yet been definitively assigned to a particular species in the database.

Potential functional bacteria for nitrate-dependent Sb(III) oxidation were investigated. The microbial community composition showed that *Oxalobacteraceae* (43.4%) and *Symbiobacteriaceae* (28.2%) dominated, followed by *Massillia* (15.4%) in the Sb(III) and nitrate amendment microcosm (Fig. S6). In addition, the species *Symbiobacteriaceae* sp. (unclassified 2), *Oxalobacteraceae* sp. (unclassified), *Massilia* sp. (unclassified), and *Bradyrhizobium* sp. (unclassified) were found to be significantly enriched in the Sb(III) and nitrate amendment as evidenced by the linear discriminant effect size (LEfSe) analysis (Fig. 2). This suggests that the unclassified bacteria may play a pivotal role in the nitrate-dependent Sb(III) oxidation.

Given the uncultured status of the functional bacteria as predicted by the 16S rRNA gene analysis and our failed isolation attempts, we carried out metagenomic sequencing followed by metagenome-assembled genomes (MAGs) analysis as an alternative approach. The putative genome (bin) of *Symbiobacteriaceae* (bin16) was successfully reconstructed, but those of *Oxalobacteraceae*, *Massilia*, and *Bradyrhizobium* failed (Table S4, Fig. S7). In addition, the bins of *Bryobacteraceae* (bin18), *Azospirillum* (bin21), *Allorhizobium* (bin14), *Pseudorhodoplanes* (bin9), *Pseudoxanthomonas* (bin24), *Pseudomonas* (bin3), *Microvirga* (bin11), and *Noviherbaspirillum* (bin1) were obtained (Table S4, Fig. S7). The reconstructed genomes serve as the foundation for the subsequent functional gene analysis.

### Genes responsible for the nitrate-dependent Sb(III) oxidation

**Figure 3:**
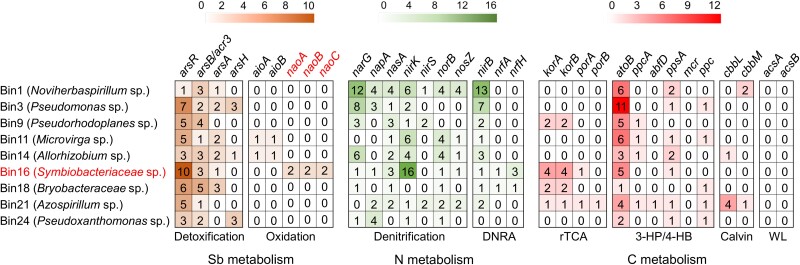
Counts of genes responsible for Sb, N, and C metabolism detected in the metagenome-assembled genomes from the microcosm of soil + Sb(III) + NO_3_^−^. The genes *narG*, *napA*, *nasA*, *nirK*, *nirS*, *norB*, and *nosZ* are responsible for denitrification; *nirB*, *nrfA*, and *nrfB* for the dissimilatory nitrate reduction to ammonium (DNRA). The genes *korA*, *korB*, *porA*, and *porB* are responsible for the reductive citrate cycle (rTCA cycle); *atoB*, *ppcA*, *abfD*, *ppsA*, *mcr*, and *ppc* for the 3-hydroxypropionate/4-hydroxybutyrate (3-HP/4-HB cycle); *cbbL* and *cbbM* for the Calvin cycle (Calvin); and *acsA* and *acsB* for the reductive acetyl-CoA pathway (Wood–Ljungdahl). “WL” refers to Wood–Ljungdahl. The genes *arsR*, *arsB*/*acr3*, *arsA*, and *arsH* are responsible for Sb(III) resistance; *aioAB* and *naoABC* for Sb(III) oxidation.

The functional genes involved in denitrification, carbon fixing, and Sb detoxification in these MAGs were analyzed. All denitrification genes were detected in the MAGs, including the NO_3_^−^ reductase gene (*narG*, *napA*, and *nasA*), the NO_2_^−^ reductase gene (*nirK*, *nirS*), the NO reductase gene (*norB*), and the N_2_O reducing gene (*nosZ*) [47] (Fig. 3). These genes are responsible for the production of gaseous substances, including N_2_, N_2_O, and NO (Fig. 1E). Genes for DNRA [48], including *nirB*, *nrfA*, and *nrfH*, were also identified (Fig. 3). Among the genes involved in carbon fixing, those associated with the 3-hydroxypropionate/4-hydroxybutyrate (3-HP/4-HB cycle), including *atoB*, *ppcA*, *abfD*, *ppsA*, *mcr*, and *ppc*, were detected in each MAG. The genes *korA*, *korB*, *porA*, and *porB* associated with the reductive citrate cycle (rTCA cycle) [49] were found in bins 9, 16, 18, and 21 (Fig. 3), suggesting their potential for autotrophic growth. In addition, the genes for Sb resistance and oxidation were observed. Specifically, Sb resistance genes, including *ars* and *acr3* for the detoxification through efflux of Sb(III) [50], were detected in all MAGs (Fig. 3). The As/Sb(III) oxidation gene *aioAB* was found in *Microvirga* (bin11) and *Rhizobium* (bin14), suggesting that these strains could probably harness the energy by coupling Sb(III) oxidation with nitrate reduction [51, 52]. However, *aioAB* was not observed in *Symbiobacteriaceae* (bin16), indicating the potential existence of hitherto unknown Sb(III) oxidases. Subsequently, we focused on the investigation of the nitrate-dependent Sb(III) oxidase present in *Symbiobacteriaceae* (bin16).

Considering enzymes responsible for catalyzing Sb redox reactions mainly belong to the DMSOR family [14, 15]. We performed our Sb(III) oxidase search within the DMSOR family. A total of 17 enzymes affiliated with the DMSOR family were found in *Symbiobacteriaceae* (bin16), including 12 previously characterized and 5 remained uncharacterized. The identified genes encode for formate dehydrogenase (FdnG), NADH-quinone oxidoreductase (NuoG), dimethyl sulfoxide reductase (DmsA), nitrate reductase (NarG), and xanthine dehydrogenase (Table S5). The five uncharacterized DMSOR were classified into two groups based on their potential functions in Sb(III) oxidation. It seems reasonable to conclude that gene0576, gene2689, and gene5022 are unlikely to be capable of oxidizing Sb(III). Gene0576 is clustered with the threonine/serine dehydratase gene, suggesting a potential involvement in amino acid metabolism. Gene2689 is located between a gene encoding xanthine dehydrogenase family protein subunit M and a gene of a (2Fe-2S)-binding protein, which suggests its involvement in xanthine metabolism [53]. Gene5022 is positioned downstream of a glucose-6-phosphate isomerase gene, which indicates a related function in glucose metabolism.

**Figure 4:**
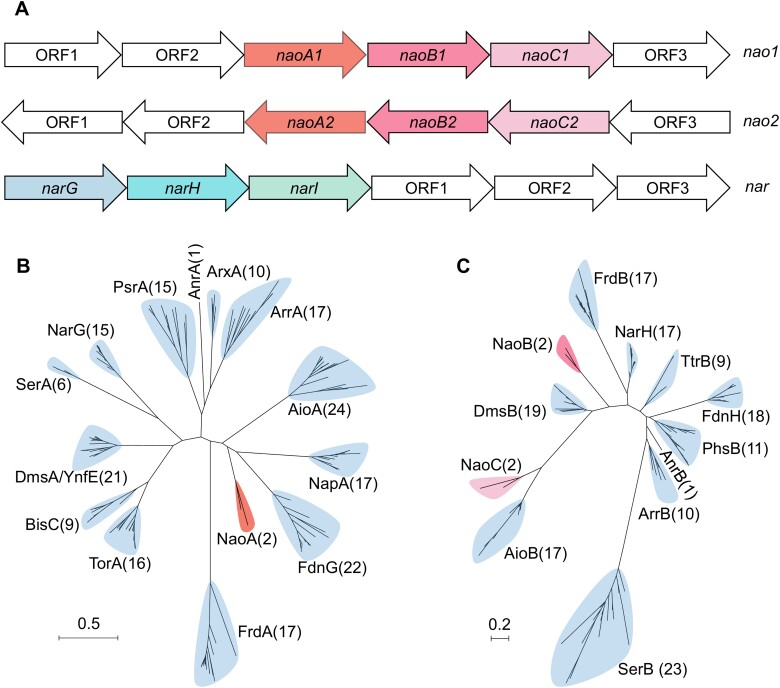
Functional genes for nitrate-dependent Sb(III) oxidation in uncultured *Symbiobacteriaceae*. Organization of *nao* and *nar* clusters possibly functioned as coupling Sb(III) oxidation with nitrate reduction (**A**). *nao1* and *nao2* cluster encoding Sb(III) oxidase, and *nar* for nitrate reductase. ORF represents the open reading frame which is annotated as hypothetical protein. Phylogenetic affiliation of the Mo-bioMGD unit NaoA (**B**), and Fe-S units NaoB and NaoC (**C**) in the unrooted trees constructed using representative sequences of the catalytic subunit, and iron–sulfur subunit of the DMSOR members. Aio, arsenite oxidase; Fdn, formate dehydrogenase; nap, periplasmic nitrate reductase; YnfF/Dms, dimethyl sulfoxide reductase; Bis, biotinsulfoxide reductase; tor, trimethylamineoxide reductase; Ser, selenate reductase; Nar, membrane-associated nitrate reductase; Arr, arsenate respiratory reductase; Psr, polysulfide reductase; Arx, arsenite oxidase; Frd, fumarate reductase; Anr, antimony reductase. Values in parentheses represent the number of sequences used. Scale bar represents the amino acid change on the branch of the length unit.

Gene0694 and gene2810, which exhibit a similar organizational trait despite their disparate locations within distinct operons, potentially function in Sb(III) oxidation. In the operon containing gene0694 (denoted as *naoA1*), the downstream gene0695 (*naoB1*) encoding the 4Fe-4S cluster and gene0696 (*naoC1*) encoding the Rieske 2Fe-2S domain display their electron transfer functionality (Table S5, Fig. 4A). Likewise, gene2810 (denoted as *naoA2*) was followed by gene2809 (*naoB2*) and gene2808 (*naoC2*) with electron transfer subunits in a distinct operon (Table S5, Fig. 4A). In addition, the hydrophobicity analysis showed that gene0694 (*naoA1*) and gene2810 (*naoA2*), and gene0695 (*naoB1*) and gene2809 (*naoB2*) encode extra-membrane hydrophilic proteins. The hydrophobic proteins are encoded by gene0696 (*naoC1*) and gene2808 (*naoC2*) with a weakly charged transmembrane helix at the N-terminal region that crosses the cytoplasmic membrane (Fig. S8). These results indicate that the Nao complexes may consist of the periplasmic proteins NaoA and NaoB which bind to the membrane by NaoC, forming a stable membrane-anchoring heterotrimeric NaoABC. Based on this typical physiological structure of DMSOR, we speculate that the NaoABC complex, in conjunction with the nitrate reductase NarGHI (Fig. 4A), may be capable of coupling Sb(III) oxidation and nitrate reduction.

### NaoABC complex represents a new subclass of the DMSOR family

The protein phylogenetic trees were constructed to gain insight into the evolutionary relationship between NaoABC and well-characterized members of the DMSOR family. The catalytic subunit NaoA constituted a discrete lineage, distinct from the other well-characterized DMSOR members, and exhibited the closest clustering with the nitrate reductase, NapA [54] (Fig. 4B). The electron transfer subunits NaoB were found to form a distinct subgroup when clustered with the 4Fe-4S subunit. The most closely related subgroup was that of the dimethyl sulfoxide reductase subunit B, DmsB [55]. NaoC with a Rieske 2Fe-2S domain diverged into a distinct evolutionary lineage and exhibited the highest similarity to the small subunit of arsenite oxidase, AioB [56, 57] (Fig. 4C). These results indicate that the NaoABC complex represents a new subclass within the DMSOR family. Despite the variance in operon configuration between NaoABC and AioAB, an evolutionary parallel was found between their catalytic components, NaoA and AioA, which are closely related to the NapA lineage. Likewise, the electron transfer modules NaoBC and AioB are evolutionarily associated with the DmsB group. This phylogenetic correspondence suggests that NaoABC and AioAB have a similar functional role in catalyzing the oxidation of Sb(III).

### NaoABC complex mediates autotrophic Sb(III) oxidation

The minimum inhibitory concentration (MIC) of Sb(III) was measured to determine appropriate concentration of Sb(III) for incubation with engineered *R. palustris*. The results showed that the MIC of Sb(III) for *R. palustris* in MSM medium was 0.5 mM, and 0.1 mM of Sb(III) had no effect on the metabolism of *R. palustris*, as indicated by similar numbers of active cells in the presence and absence of 0.1 mM Sb(III) (Fig. S9). Therefore, we selected 0.1 mM of Sb(III) for the incubation experiment with *R. palustris* strains.

To verify that *naoABC* is responsible for autotrophic Sb(III) oxidation under denitrification conditions, two *nao* clusters were heterologously expressed in carbon dioxide–fixing *R. palustris*, which naturally lacks the reported Sb(III) oxidation genes *aioAB* and *anoA*. In the incubation with only nitrate (control condition), Nao1 and Nao2 exhibited no Sb(III) oxidation, and ^13^C incorporation, similar to that of control cells (*R. palustris*-pCE2TA) (Fig. S10A, C). However, in the incubation with Sb(III) and nitrate (autotrophic condition), the insertion of *nao1* and *nao2* apparently increased dissolved Sb(V) (1.25 for Nao1 and 1.75 μM for Nao2) (Fig. S10A), ^13^C assimilation (Fig. S10C–J), and the cell numbers (from 1.04 × 10^5^ to 4.20 × 10^5^ for Nao1, and from 1.01 × 10^5^ to 5.86 × 10^5^ cells/mL for Nao2) compared to control cells (Fig. S10B). These results indicate that the NaoABC complex is capable of autotrophic oxidation of Sb(III) under denitrification conditions, with *nao2* cluster serving as the main functional determinant.

**Figure 5:**
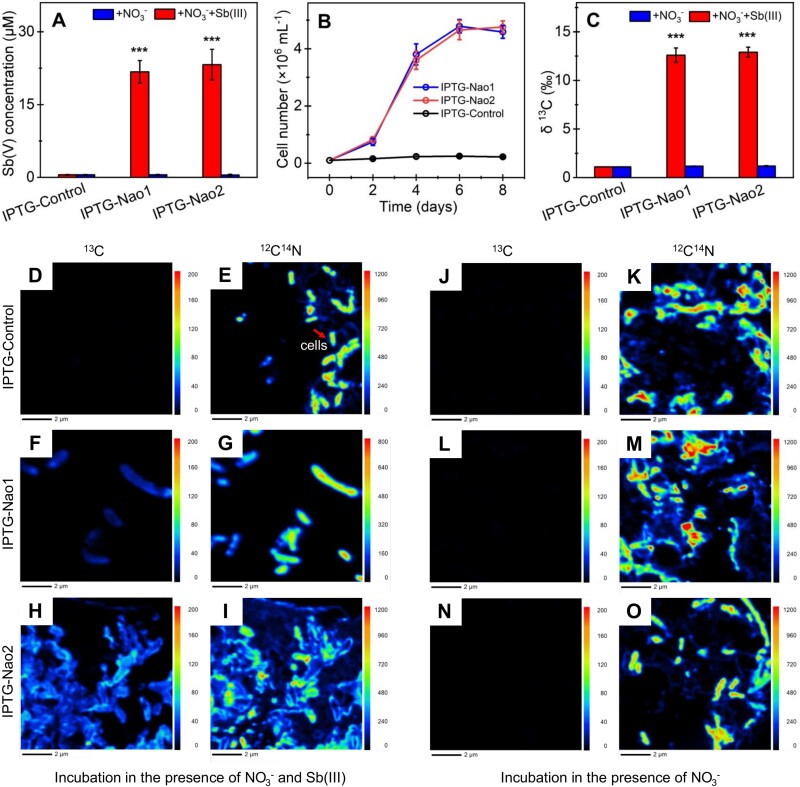
Autotrophic oxidation of Sb(III) in recombinant *R. palustris* enhanced by IPTG. The amount of Sb(III) oxidation (**A**), cell number monitored over time (**B**), ^13^C incorporation by the isotope ratio mass spectrometry analysis (**C**), nano-SIMS images of ^13^C (**D**, **F**, and **H**) and ^12^C^14^N (as indicator of biomass) (**E**, **G**, and **I**) in the incubation with Sb(III) and nitrate (autotrophic condition), and images of ^13^C (**J**, **L**, and **N**) and ^12^C^14^N (**K**, **M**, and **O**) in the incubation with only nitrate (control condition). IPTG-control stands for *R. palustris-*pCE2TA, IPTG-Nao1 for *R. palustris*-pCE2TA*-nao1*, and IPTG-Nao2 for *R. palustris*-pCE2TA-*nao2* induced by IPTG. Error bars represent the standard deviations of means of triplicates. Scale bars, 2 μm. Vertical scale bars on the right indicate ^13^C or ^12^C^14^N atom percent. Asterisks indicate IPTG-Nao1 and IPTG-Nao2 significantly differ from IPTG-control (^*^^*^^*^P < .001).

The Sb(III) oxidation capacity of the *nao* genes in the engineered *R. palustris* was enhanced by inducing the overexpression of NaoABC complex through the addition of 0.2 mM IPTG. The overexpressed Nao proteins significantly increased the dissolved Sb(V) from 1.25–1.75 to 21.75–23.24 μΜ (Fig. 5A), and increased cell numbers from 4.20 × 10^5^–5.86 × 10^5^ to 4.59 × 10^6^–4.76 × 10^6^ cells/mL (Fig. 5B). Additionally, the ratio of ^13^C increased from 1.7–2.2 to 12.6–12.9‰ (Fig. 5C). Nano-SIMS imaging provided further corroboration of these findings by visualizing the assimilation of ^13^C. As anticipated, Fig. 5K, M, and O demonstrated that the biomass was present in IPTG-Nao1 and IPTG-Nao2, with no discernible ^13^C, akin to that observed in IPTG-control cells within the control condition with solely nitrate (Fig. 5J, L, and N). Conversely, in the incubation with Sb(III) and nitrate, a comparable signal of ^13^C was observed in both IPTG-Nao1 and IPTG-Nao2, but no ^13^C was detected in IPTG-control cells (Fig. 5D, F, and H), and ^12^C^14^N was employed as an indicator of biomass (Fig. 5E, G, and I). Different from a higher function of Nao2 than Nao1 observed above, the overexpressed Nao1 and Nao2 showed comparable capabilities, which is probably attributed to the induction of similar levels of protein expression. These pieces of evidence demonstrate that *nao* genes enable *R. palustris* to oxidize Sb(III) under denitrification conditions, producing the energy required for ^13^C incorporation and autotrophic growth. Given the identification of carbon-fixing pathways in the functional microbe *Symbiobacteriaceae* (bin16) (Fig. 3), we speculate that *Symbiobacteriaceae* (bin16) is capable of utilizing Sb(III) as a primary electron donor for chemolithoautotrophic growth.

## Discussion

In this study, a new Sb(III) oxidase, designated as NaoABC, is discovered in an uncultured *Symbiobacteriaceae* species based on the application of metagenomics, heterologous expression, ^13^C labeling, and nano-SIMS techniques. The NaoABC enzyme coupled with nitrate reductase NarGHI forms an energy-generating respiratory chain for nitrate-dependent Sb(III) oxidation. This respiratory chain represents a new enzymatic link between the Sb and N cycles, which significantly influences the behavior and fate of Sb under anoxic conditions.

Similar to oxygen, nitrate is commonly employed as an electron acceptor to generate the energy for the growth of autotrophic microorganisms [58]. This autotrophic denitrification is often coupled with the oxidation of other elements, including C [59], S [60, 61], Fe [62], and As [40]. Here, multiple lines of evidence indicate that Sb(III) can serve as an electron donor for autotrophic denitrification. In fact, previous studies have reported the existence of a nitrate-dependent Sb(III) oxidation pathway. The functional bacteria were isolated, including *H. taeniospiralis* IDSBO-1, *Ensifer* sp. NLS4 [10], and *Sinorhizobiu*m sp. GW3 [11]. These results suggest that the pathway of nitrate-dependent Sb(III) oxidation is widely used by microbes. This conclusion provides a plausible explanation for the occurrence of high Sb(V) in anoxic environments, including subsurface groundwater and rice paddy soils [63]. Moreover, the progression of nitrate-dependent Sb(III) oxidation could potentially reduce the accumulation of Sb in agricultural ecosystems. This is because crops tend to take up Sb(III) over Sb(V). Therefore, the application of nitrogen fertilization and the subsequent nitrate formation may serve to minimize Sb toxicity.

*Symbiobacteriaceae*-related microbes are typically uncultured, and commonly found in groundwater and agricultural soils [64]. The presence of some *Symbiobacteriaceae* members has been documented in Sb-contaminated rice soils [65], yet their role in Sb transformation has not been elucidated. The present study reveals that *Symbiobacteriaceae* species represent new organisms with the capacity to couple autotrophic Sb(III) oxidation with nitrate reduction, thereby expanding the diversity of Sb(III) oxidizing microbial lineages. The potential to manipulate these *Symbiobacteriaceae* species represents an innovative strategy for the *in situ* remediation of Sb pollution. However, before drawing this conclusion, more related work stimulating the growth of *Symbiobacteriaceae* species in Sb-contaminated soil is necessary.

The enzyme AioAB was previously proposed to be the primary catalyst for the oxidation of Sb(III) [2]. Nevertheless, the distinctive NaoABC complex was observed to mediate this process in *Symbiobacteriaceae* species. Despite both AioAB and NaoABC belonging to the DMSOR family, they display distinct structural organization and evolutionary history (Fig. 4). The identification of a new Sb(III) oxidase, NaoABC, is a notable discovery as it represents a hitherto unidentified pathway of autotrophic Sb(III) oxidation. Moreover, *naoA* can be used as a molecular probe complementary to *aioA*, potentially facilitating the discovery of diverse functional species involved in Sb(III) oxidation. This deeper understanding in microbial ecology is essential for the development of innovative strategies to bio-remediate environments contaminated with Sb.

**Figure 6:**
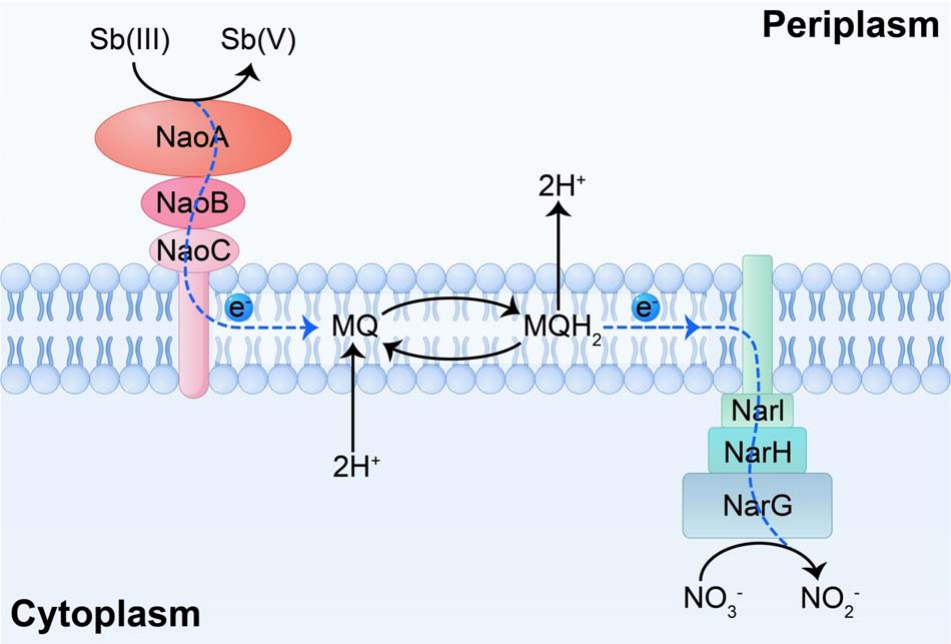
Schematic diagram for the enzymatic mechanism of nitrate-dependent Sb(III) oxidation in uncultured *Symbiobacteriaceae* species. Sb(III) donates electrons and is oxidized by the NaoABC complex. Subsequently, these electrons are transferred to the quinone pool. Finally, the reduced quinols convey the electrons to the membrane-bound nitrate reductase complex NarGHI, where nitrate is reduced. In this Nao-quinol-Nar chain, the transfer of electrons from Sb(III) to nitrate is coupled with the generation of a proton motive force across the cytoplasmic membrane.

The electron transfer pathway of nitrate-dependent Sb(III) oxidation follows a classical respiratory chain as previously described [66]. The schematic diagram elucidates the electron flow from Sb(III) to nitrate (Fig. 6). Specifically, Sb(III) is oxidized by NaoABC complex, which subsequently links the electron transfer chain via quinone reduction. The quinol pool, which is capable of lipid diffusion, then conveys the electrons to a membrane-anchored NarGHI nitrate reductase complex, as observed in the bin16 genome (Fig. 4A), where nitrate is reduced to nitrite. In this Nao-quinol-Nar chain, the electron transfer from Sb(III) to nitrate is coupled to the generation of a proton motive force across the cytoplasmic membrane [67], thereby generating energy for cellular growth.

Metagenomics, a culture-independent technique, represents a powerful tool for the discovery of innovative genes and enzymes from uncultured microorganisms. In this study, we employed a combination of heterologous expression and isotope technology to identify a subclass of the DMSOR family with the capacity to couple Sb(III) oxidation with nitrate reduction. Indeed, over 300 new enzymes including cellulose, hemicellulase, chitinase, and oxidoreductase have been discovered by mining the metagenomic data from 2014 to 2017 [68]. For example, a new CRISPR-Cas system in uncultured microbes was identified from metagenomic datasets of groundwater, sediment, acid mine drainage biofilms, and soil [69]. A bifunctional xylanase/β-glucosidase that enhances ethanol production was selected by the metagenomic analysis [70]. Moreover, MAGs facilitate the elucidation of new metabolic pathways. A complete nitrification pathway (comammox) was elucidated in *Nitrospira* sp. reconstructed from the metagenomic data of an enriched microbial biofilm, fundamentally changing the understanding of the nitrogen cycle [71]. It is indisputable that metagenomics will assume an increasingly important role in the discovery of valuable genes and metabolic pathways from uncultured organisms. This will contribute to a more profound comprehension of microbial metabolism and open up new avenues for biotechnological applications.

## Supplementary Material

SI_wrae204

## Date availability

Details of MSM components, passage experiment of microcosms, the 16S rRNA gene amplification and sequencing, soluble Sb(III) and Sb(V) analyses, MIC detection, the cloning of NarGHI to *R. palustris*, cell number determination, and supplementary tables and figures are shown in SI. The 16S rRNA gene amplicon and metagenomic reads were deposited in the NCBI database under accession numbers of PRJNA862322 and PRJNA862653, respectively.
