# Supplementary material for: Nitrate-dependent antimony oxidase in an uncultured Symbiobacteriaceae member
Source: ISME J. 2024 Oct 16;18(1):wrae204. doi: 10.1093/ismejo/wrae204 (PMC11521347; doi:10.1093/ismejo/wrae204)
Supplement: SI_wrae204 [file si_wrae204.docx]

Supplementary Information

Nitrate-dependent antimony oxidase in an uncultured *Symbiobacteriaceae* member

Liying Wang^1^, Zhipeng Yin^1^, Wei Yan^1^, Jialong Hao^2^, Fei Tian^3^, Jianbo Shi^1,4,5*^

^1^State Key Laboratory of Environmental Chemistry and Ecotoxicology, Research Center for Eco-Environmental Sciences, Chinese Academy of Sciences, Beijing, China

^2^ Key Laboratory of Earth and Planetary Physics, Institute of Geology and Geophysics, Chinese Academy of Sciences, Beijing, China

^3^CAS Engineering Laboratory for Deep Resources Equipment and Technology, Institute of Geology and Geophysics, Chinese Academy of Sciences, Beijing, China

^4^School of Environment, Hangzhou Institute for Advanced Study, University of Chinese Academy of Sciences, Hangzhou, China

^5^MOE Key Laboratory of Groundwater Quality and Health, School of Environmental Studies, China University of Geosciences, Wuhan, China

***Corresponding author:** Dr. Jianbo Shi, State Key Laboratory of Environmental Chemistry and Ecotoxicology, Research Center for Eco-Environmental Sciences, Chinese Academy of Sciences, 18 Shuangqing Road, Haidian District, Beijing, 100085, China

E-mail: [jbshi@rcees.ac.cn](file:///F:\Study\文档\geothite-ENR-bacteria\cyjing@rcees.ac.cn)

**Short running title:** Antimony oxidase in *Symbiobacteriaceae*

**Supplementary Methods**

**Mineral salts medium (MSM)**

The MSM contains the following ingredients (grams per liter of deionized H_2_O): 1.9 g of NaHCO_3_, 0.2 g of KH_2_PO_4_, 0.25 g of NH_4_Cl, 0.4 g of MgCl_2_, 0.5 g of KCl, 1.0 g of NaCl, 0.1 g of CaCl_2_, 10 mL vitamin solution, and 1 mL SL-10 trace elements solution. The pH of the medium was adjusted to 7.2 with HCl.

The SLM contains the following ingredients (grams per liter of deionized H_2_O): 2.6 g sodium lactate, 0.4 g NH_4_Cl, 0.5 g KHPO_4_, 0.1 g MgSO_4_, 0.1 g yeast extract, 10 mL vitamin solution, and 1 mL SL-10 trace elements solution. The pH of the medium was adjusted to 7.2 with HCl.

**Passage experiment of microcosms**

In order to reduce abiotic Sb(III) oxidation, soil microcosms (1^st^ generation) were passaged. Briefly, at the end of the soil incubations, 2 mL supernatant from the serum bottle was extracted and mixed with 100 mL fresh MSM medium supplemented with 1 mM Sb(III) and 10 mM nitrate to initiate the passage. The anoxic passage was performed every 8 days in a glovebox (100% N_2_). Periodically, 1 mL of the suspension was sampled using a sterile needle to determine the concentrations of Fe(II), Sb(V), Sb(III), NO_3_^-^, and NO_2_^-^.

**Amplification and sequencing of the 16S rRNA gene**

The PCR amplification of 16S rRNA gene is performed as follows: initial denaturation at 95°C for 10 min, followed by 30 cycles of denaturing at 95°C for 30 s, annealing at 55°C for 30 s, and extension at 72°C for 1 min, and final extension at 72°C for 10 min. The PCR mixtures contain 5×TransStart FastPfu buffer 4 μL, 2.5 mM dNTPs 2 μL, forward primer (5 μM) 0.8 μL, reverse primer (5 μM) 0.8 μL, TransStart FastPfu DNA Polymerase 0.4 μL, template DNA 10 ng, and finally ddH_2_O up to 20 μL. The PCR product is extracted from 1.5% agarose gel and purified using the AxyPrep DNA Gel Extraction Kit (Axygen Biosciences, USA) according to manufacturer's instructions and quantified using Quantus Fluorometer (Promega, USA).

The purified amplicons were pooled in equimolar amounts and paired-end sequenced on an PE300/PE250 platform (Illumina, USA) according to the standard protocols by Majorbio Bio-Pharm Technology Co. Ltd. (Shanghai, China).

The resulting sequences were quality filtered with fastp (v0.19.6) [1] and merged with FLASH (v1.2.11) [2]. Then the high-quality sequences were denoised using the DADA2 [3] plugin in the QIIME2 [4] (v2022.2) pipeline with recommended parameters, which obtains single nucleotide resolution based on error profiles within samples. DADA2 denoised sequences are usually called amplicon sequence variants (ASVs). Taxonomic assignment of ASVs was performed using the Naive Bayes consensus taxonomy classifier implemented in QIIME2 and the 16S rRNA database SILVA (v138).

Bioinformatic analysis was carried out using the Majorbio Cloud platform (<https://cloud.majorbio.com>). Based on the ASVs information, rarefaction curves and alpha diversity including Shannon and Sobs index were calculated with Mothur (v1.30.1) [5]. Linear discriminant analysis (LDA) effect size (LEfSe) (http://galaxy.biobakery.org/) was performed to identify the significantly abundant bacterial taxa among the different groups (LDA score > 3, *P* < 0.05).

**Soluble Sb(III) and Sb(V) analyses**

The concentrations of Sb(III) and Sb(V) were determined using HPLC coupled to atomic fluorescence spectrometry (AFS). For Sb speciation analysis, an anion exchange column (PRP-X100, 4.1×250 mm, 10 μm; Hamilton) was used. The mobile phase was 10 mM EDTA, 1 mM potassium hydrogen phthalate at a flow rate of 1.0 mL/min. Following the separation of the different Sb species, online hydride generation was achieved by reaction with 1.5% wt. KBH_4_ and 7% wt. HCl. The hydride was atomized in a hydrogen flame and the fluorescence signal for each Sb species was recorded using an AFS-8800 spectrometer (AFS, Jitian, China) [6]. The detection limits were 1.2 μg/L for Sb(V) and 0.6 μg/L for Sb(III). Samples were filtered through a 0.22 μm membrane filter prior to Sb determination.

**The minimum inhibitory concentration (MIC) detection**

To determine the MIC of Sb(III), *R.* [*palustris*](https://www.so.com/link?m=bI9LsD8hToVzF%2FGxejIC8zIvvtJYcISPhieA3a2vZTY06yT9pBPMph5mr6Mb9IYjHTZ8X2hCZrwwXuNdt0Q9OFhfYJo%2F8kFWzuAqMZWp%2FbErOH5HD11flEdU5hCFMapc8VmqejdRLRO4Qs2j%2FKP60qoUfjDzDXambKxaWoKbIYQUxjQ8PsAOv12b53t2OH9Tmtw7izQ8xNfseYBi73bfpAWBZhtF4mdfxcYNLakra0diaYHe6nPNNCYFU4xErW47DbnePmRLFhDs%3D) was incubated in MSM medium amended with different concentrations of Sb(III) (0, 0.1, 0.3, 0.5, and 1.0 mM) for 24 h at 30^o^C. Periodically, cultures were sampled and analyzed for cell number.

**Cloning NarGHI to *R.*** [***palustris***](https://www.so.com/link?m=bI9LsD8hToVzF%2FGxejIC8zIvvtJYcISPhieA3a2vZTY06yT9pBPMph5mr6Mb9IYjHTZ8X2hCZrwwXuNdt0Q9OFhfYJo%2F8kFWzuAqMZWp%2FbErOH5HD11flEdU5hCFMapc8VmqejdRLRO4Qs2j%2FKP60qoUfjDzDXambKxaWoKbIYQUxjQ8PsAOv12b53t2OH9Tmtw7izQ8xNfseYBi73bfpAWBZhtF4mdfxcYNLakra0diaYHe6nPNNCYFU4xErW47DbnePmRLFhDs%3D)

To characterize the function of NaoABC which is responsible for the coupling to NO_3_^-^ reduction, the full length of the NarGHI cluster (6.9 kb) was amplified containing its own promoter and the continuous genes of *narGHI*. The genomic DNA of the soil+Sb(III)+NO_3_^-^ treatment on day 8 was used as temple and the primers used were listed in Table S2. The PCR products were cloned into plasmid pBBR1, resulting in the recombinant plasmid pBBR1*-nar*. Then, the recombinant plasmid was transformed into the carbon fixing strain *R.* [*palustris*](https://www.so.com/link?m=bI9LsD8hToVzF%2FGxejIC8zIvvtJYcISPhieA3a2vZTY06yT9pBPMph5mr6Mb9IYjHTZ8X2hCZrwwXuNdt0Q9OFhfYJo%2F8kFWzuAqMZWp%2FbErOH5HD11flEdU5hCFMapc8VmqejdRLRO4Qs2j%2FKP60qoUfjDzDXambKxaWoKbIYQUxjQ8PsAOv12b53t2OH9Tmtw7izQ8xNfseYBi73bfpAWBZhtF4mdfxcYNLakra0diaYHe6nPNNCYFU4xErW47DbnePmRLFhDs%3D) by chemical transformation, acquiring the recombinant *R.* [*palustris*](https://www.so.com/link?m=bI9LsD8hToVzF%2FGxejIC8zIvvtJYcISPhieA3a2vZTY06yT9pBPMph5mr6Mb9IYjHTZ8X2hCZrwwXuNdt0Q9OFhfYJo%2F8kFWzuAqMZWp%2FbErOH5HD11flEdU5hCFMapc8VmqejdRLRO4Qs2j%2FKP60qoUfjDzDXambKxaWoKbIYQUxjQ8PsAOv12b53t2OH9Tmtw7izQ8xNfseYBi73bfpAWBZhtF4mdfxcYNLakra0diaYHe6nPNNCYFU4xErW47DbnePmRLFhDs%3D)-*nar*.

**Determination of cell number**

Cell number was measured using a viable count method. Cultures were serially diluted and plated on solid SLM medium for incubation for 4 days. Colony forming units (CFU) were then enumerated to represent cell number.

**Supplementary Figures**

**
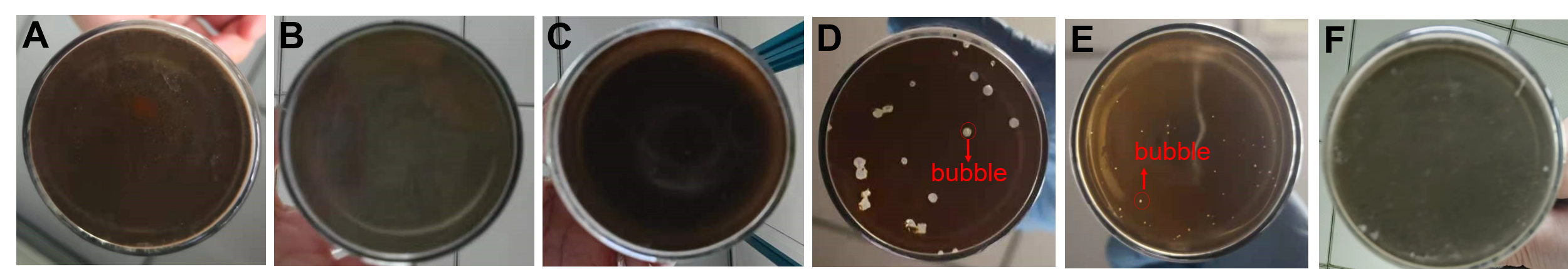
**

**Figure S1. The phenomenon of gas bubble formation during anoxic incubation.** Incubation of negative control soil (**A**), soil (**B**), soil+Sb(III) (**C**), soil+Sb(III)+NO_3_^-^ (**D**), soil+NO_3_^-^ **(E**), and soil+Sb(III)+NO_3_^-^+azide **(F)**. Microbial denitrification occurred in the presence of NO_3_^-^. The addition of Sb(III) clearly facilitated the denitrification process, indicating the possibility of a coupling between the oxidation of Sb(III) and the reduction of NO_3_^-^.

**
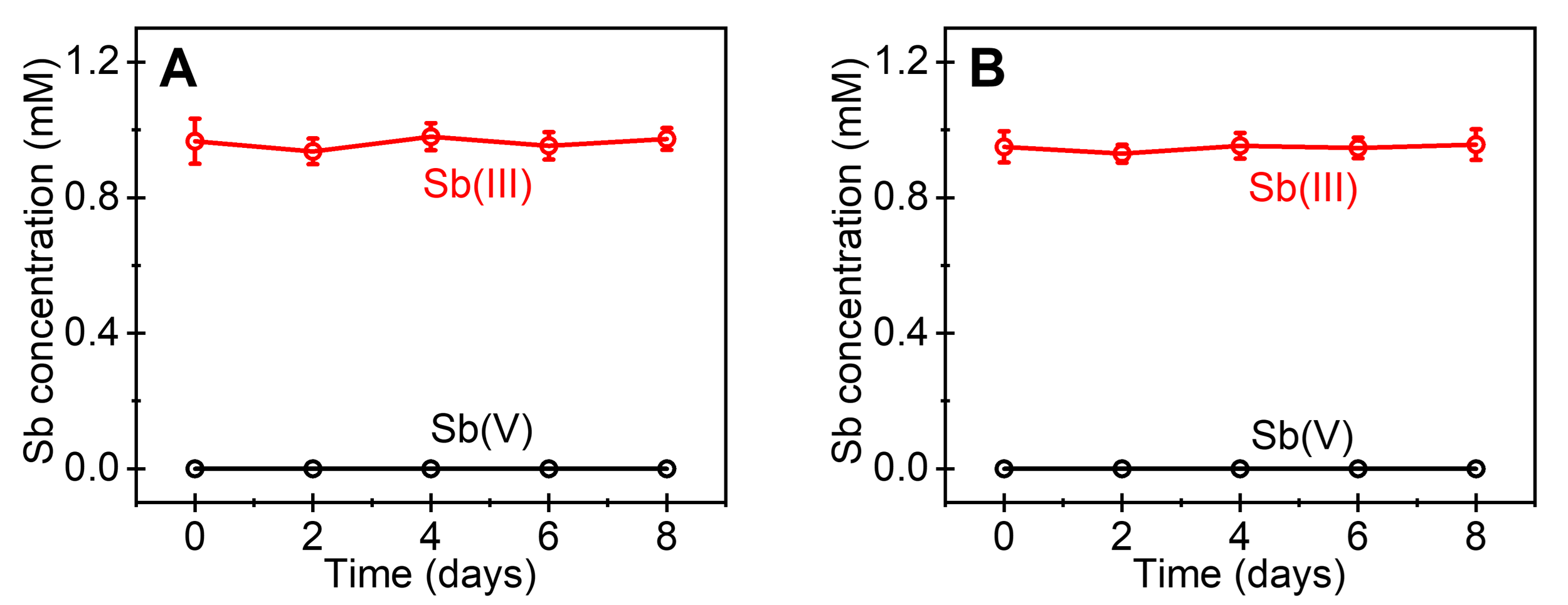
**

**Figure S2. Abiotic Sb(III) oxidation.** Oxidation of Sb(III) with FeCl_3_ (**A**) and nitrite (**B**). 1 mM Sb(III) was added to MSM medium containing 1 mM FeCl_3_ or 10 mM nitrite, and the mixture was incubated under anoxic conditions for 8 days. Error bars correspond to standard deviations of triplicate means.

**
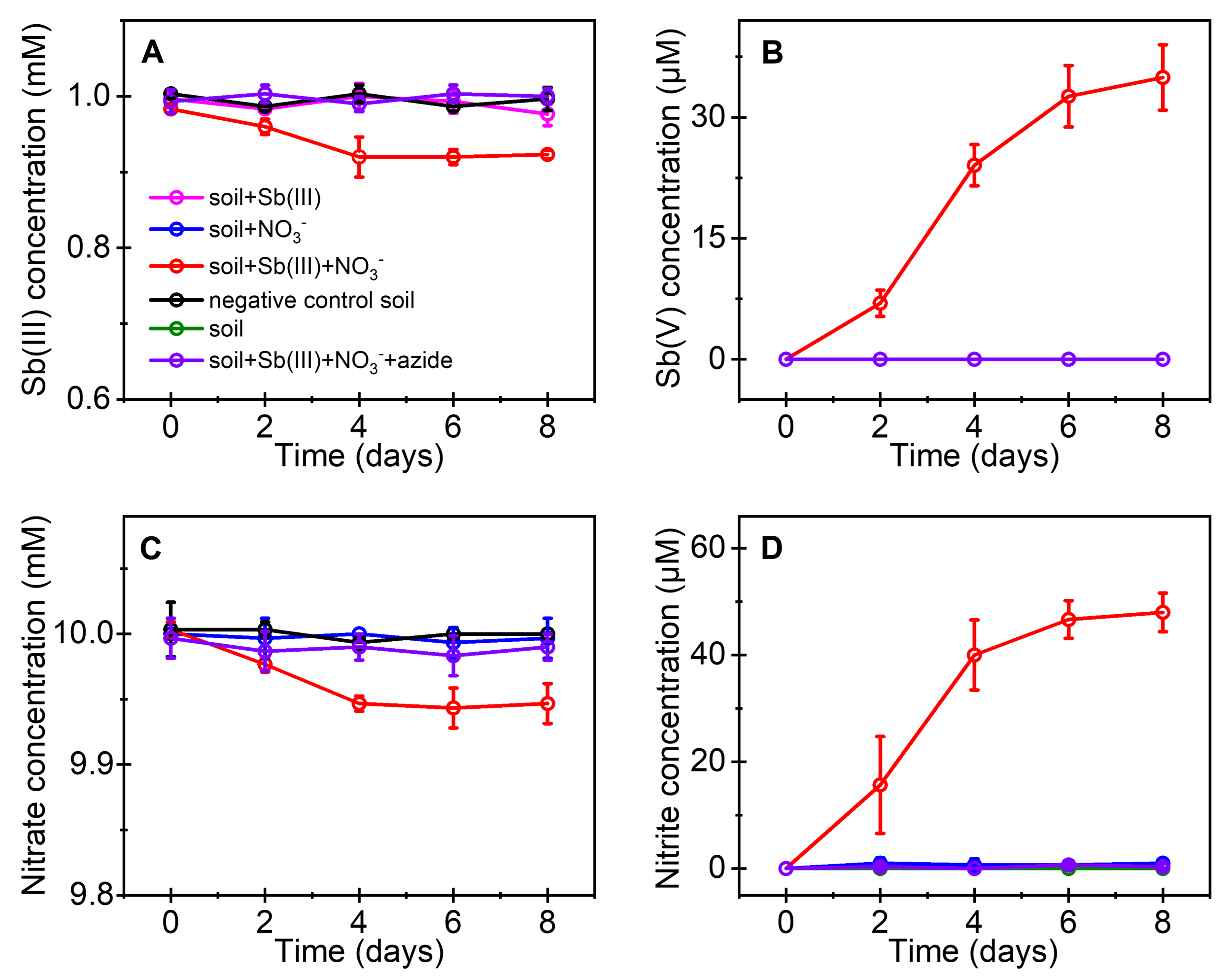
**

**Figure S3. The detection of microbial Sb(III) oxidation coupled with NO_3_^-^ reduction in the 3^rd^ generation culture.** Time dependent concentrations of dissolved Sb(III) (**A**), Sb(V) (**B**), NO_3_^-^ (**C**), and NO_2_^-^ (**D**) in the microcosm of soil+Sb(III), soil+NO_3_^-^, soil+Sb(III)+NO_3_^-^, negative control soil, soil, and soil+Sb(III)+NO_3_^-^+azide. Error bars correspond to standard deviations of triplicate means.

**
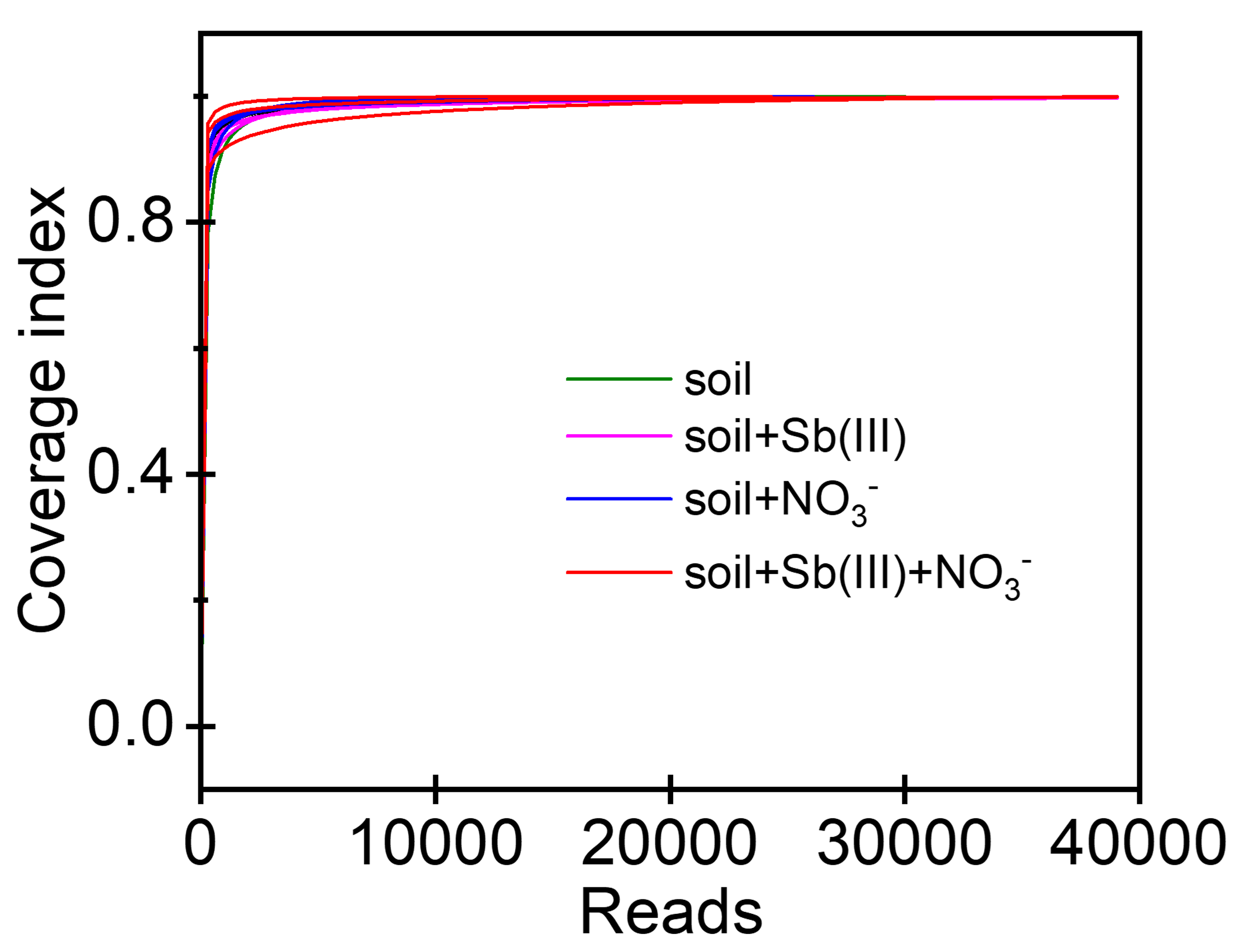
**

**Figure S4. Rarefaction curves generated from the ASVs of the incubation samples.** The coverage of the samples was high, suggesting that the depth of sequencing was sufficient for the study.

**
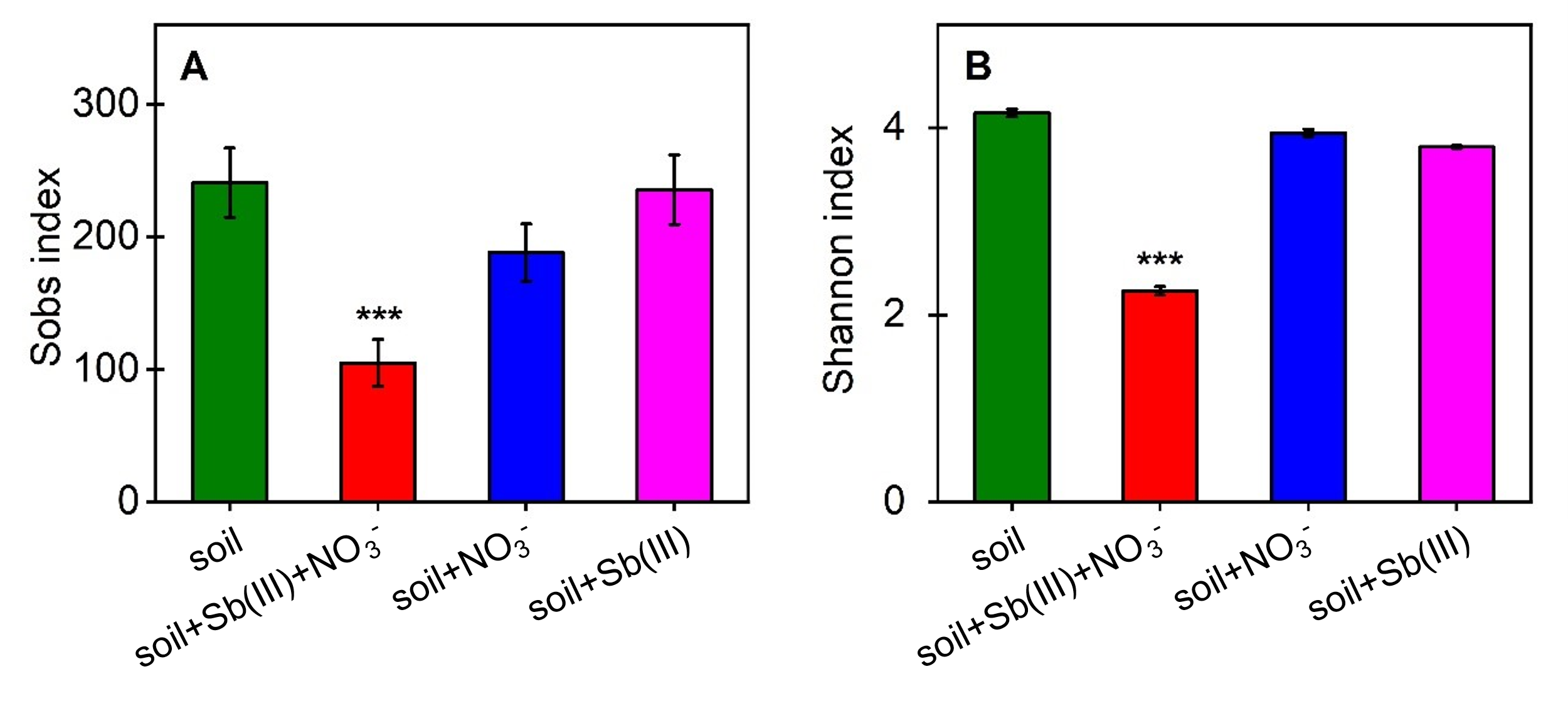
**

**Figure S5. Alpha diversity of the incubation samples at genus and species level.** Sobs (**A**) and Shannon index (**B**) indicate that Sb(III) and nitrate amendment may enrich the functional bacteria responsible for nitrate-dependent Sb(III) oxidation. Asterisks indicate that incubation of soil+Sb(III)+NO_3_^-^ and soil+NO_3_^-^ was significantly different from soil (****P* < 0.001).

**
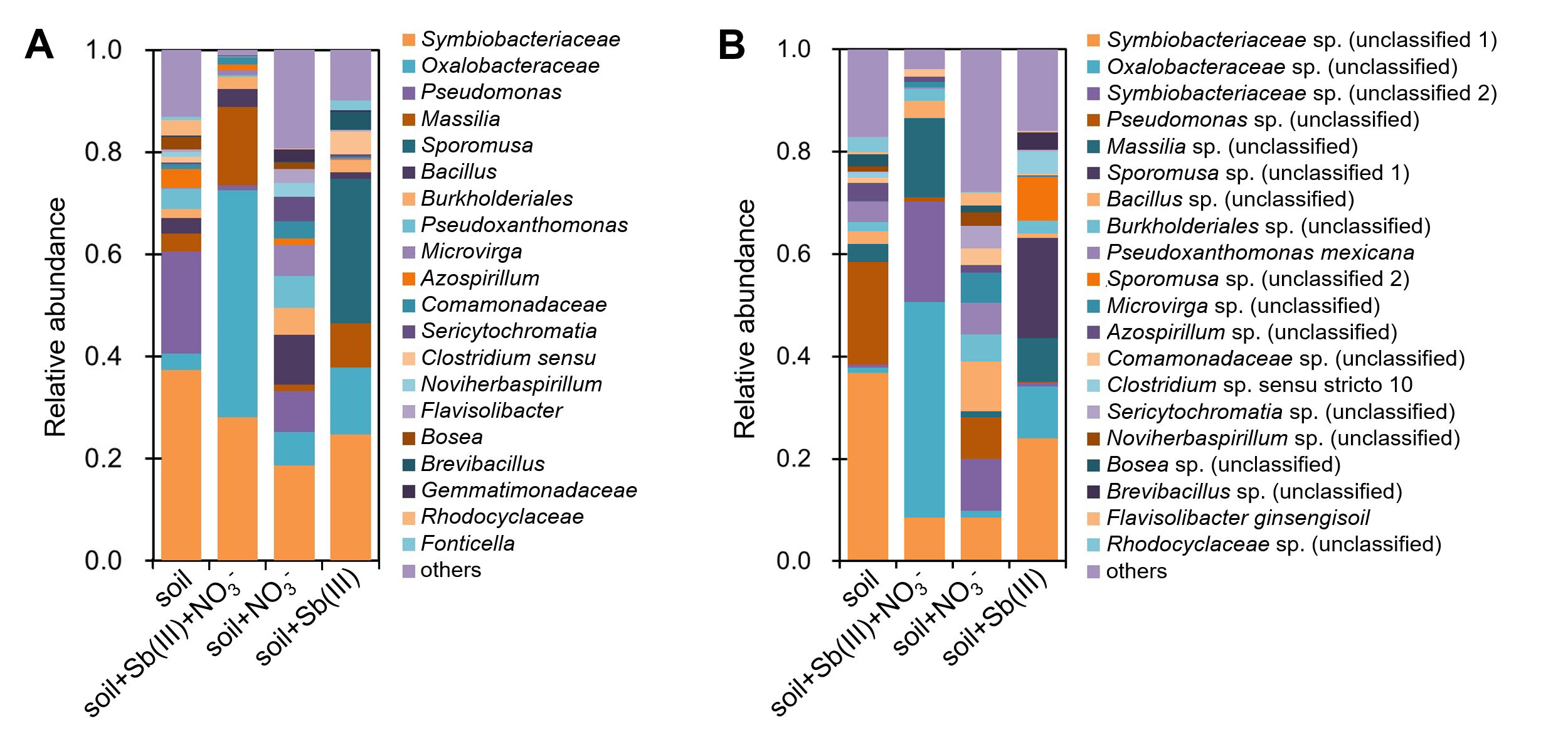
**

**Figure S6. Bacterial composition of the four incubations based on the 16S rRNA gene analysis.** Bacterial composition at genus (**A**) and species level (**B**). *Symbiobacteriaceae* sp. (unclassified 2), *Oxalobacteraceae* sp. (unclassified), and *Massilia* sp. (unclassified) were enriched in the soil+Sb(III)+NO_3_^-^ microcosm. The term “unclassified” refers to microbial strains that have not yet been definitively assigned to a particular species in the database.

**
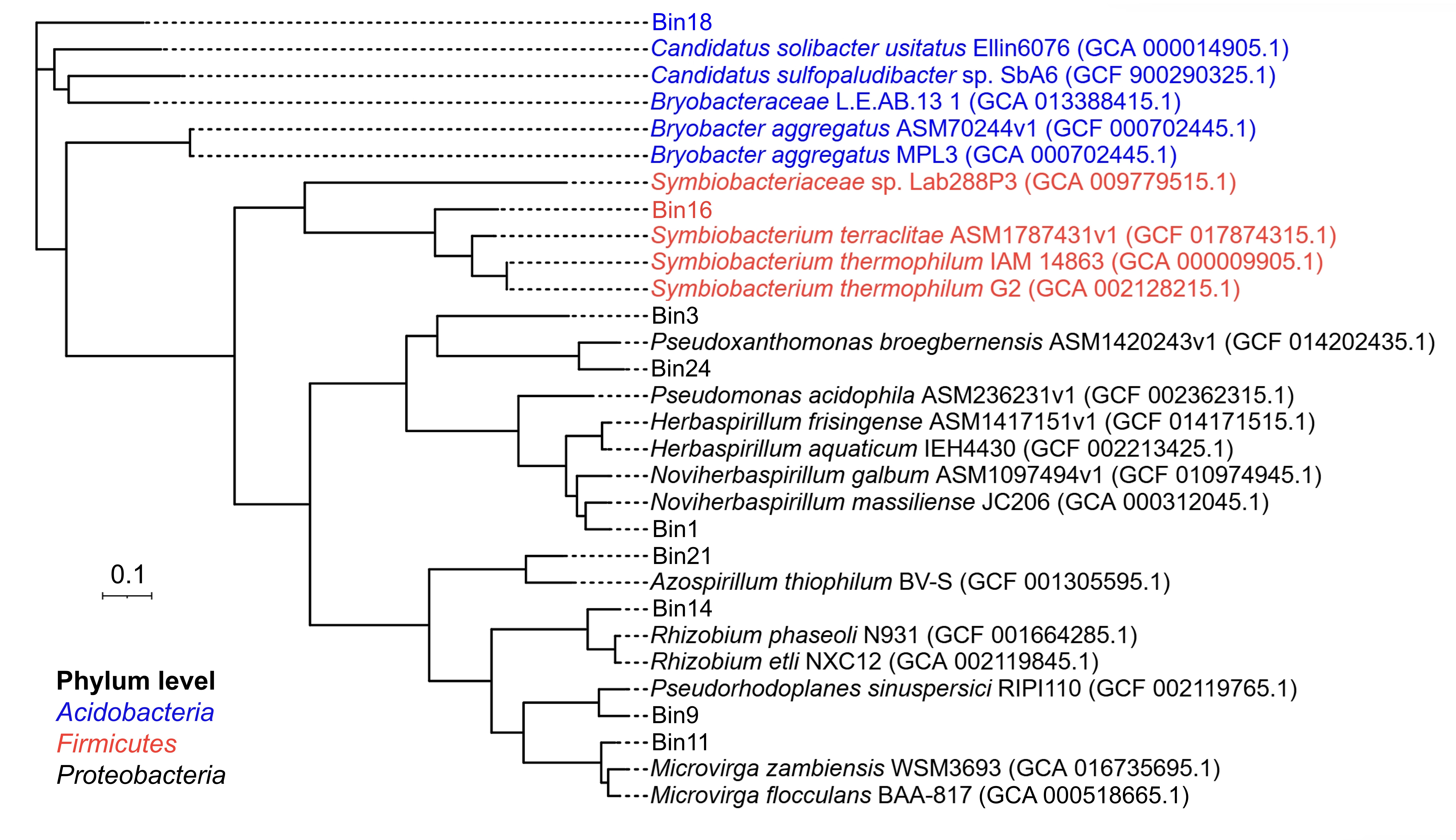
**

**Figure S7**. **Phylogenetic affiliations of the metagenome assembled genomes (MAGs).** Phylogenetic tree of the reconstructed bins and associated species, constructed using protein sequences by approximate maximum likelihood method in GTDB-Tk software. Blue strains belong to the phylum *Acidobacteria*, red strains belong to the phylum *Firmicutes*. 0.1 represents a genetic variation of 10% in the genome sequence on the branch of this length unit.

**
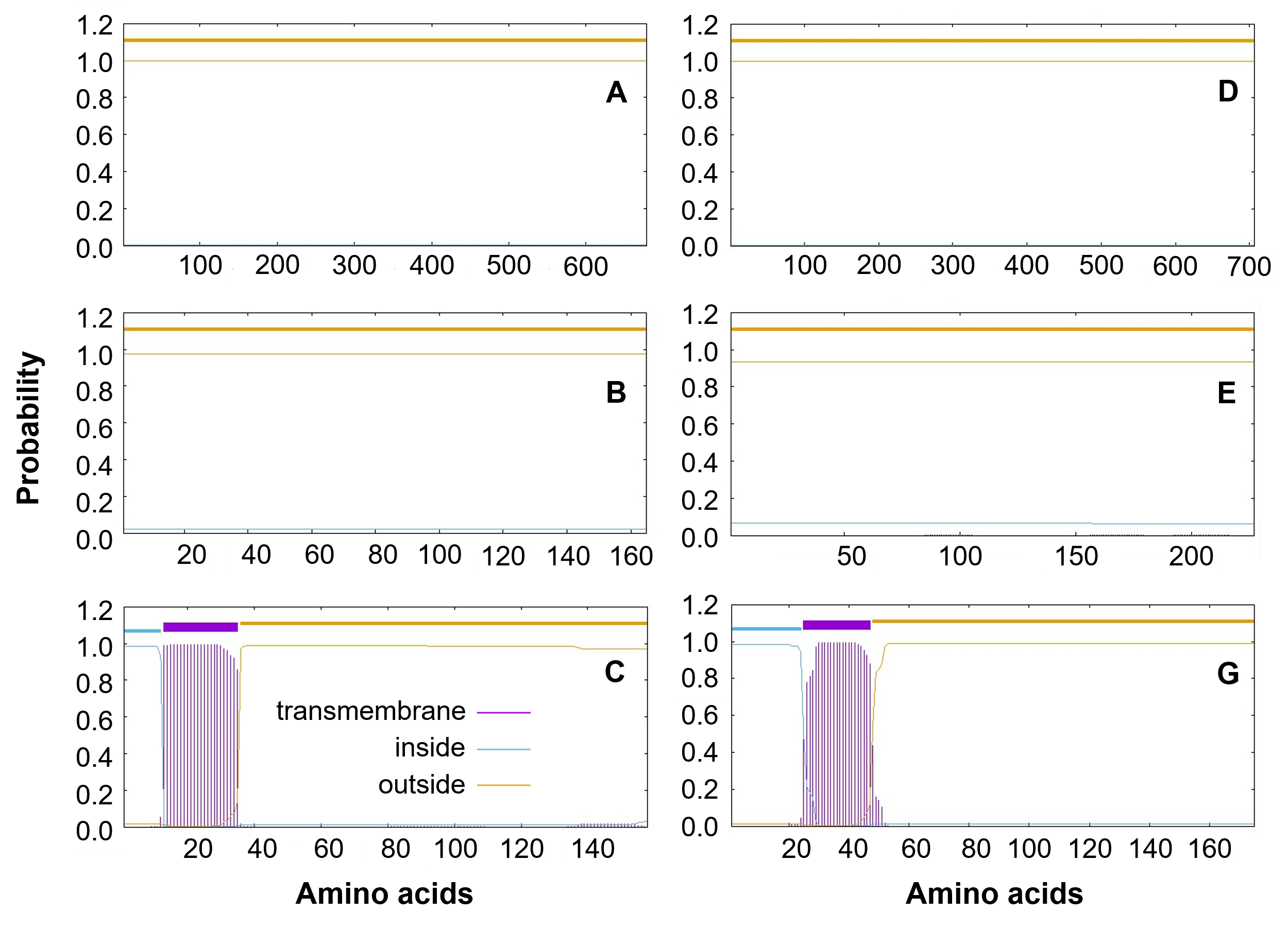
**

**Figure S8**. **Hydrophobicity analysis of the Nao subunits.** Subunit of NaoA1 (**A**), NaoB1 (**B**), NaoC1 (**C**), NaoA2 (**D**), NaoB2 (**E**), and NaoC2 (**G**). The NaoA and NaoB subunits were predicted to be extramembrane hydrophilic proteins, and NaoC was predicted to have a charged transmembrane helix at the N-terminus that could cross the cytoplasmic membrane.

**
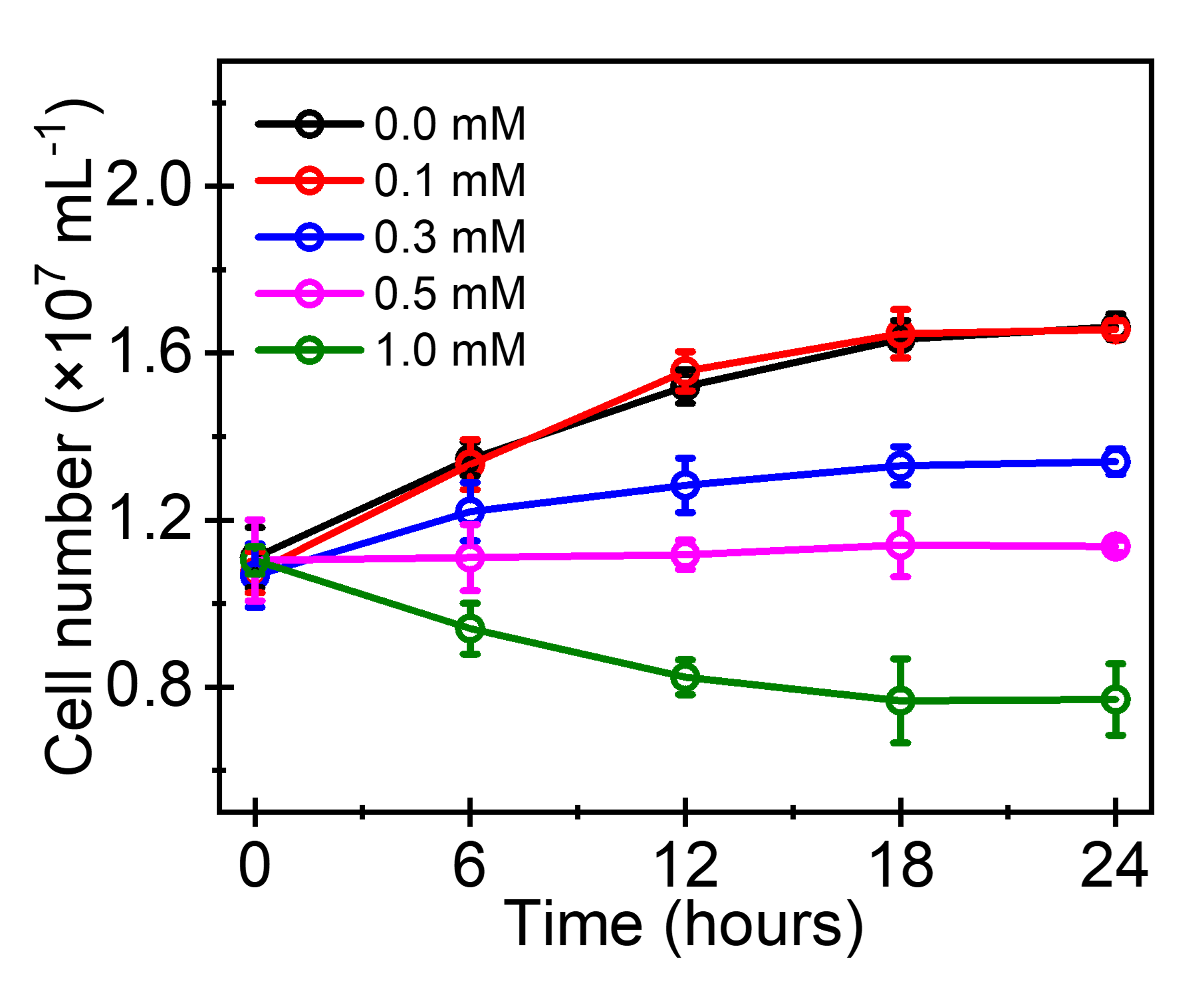
**

**Figure S9. Minimum inhibitory concentration of Sb(III) for *R. palustris*.** Cell number of *R.* [*palustris*](https://www.so.com/link?m=bI9LsD8hToVzF%2FGxejIC8zIvvtJYcISPhieA3a2vZTY06yT9pBPMph5mr6Mb9IYjHTZ8X2hCZrwwXuNdt0Q9OFhfYJo%2F8kFWzuAqMZWp%2FbErOH5HD11flEdU5hCFMapc8VmqejdRLRO4Qs2j%2FKP60qoUfjDzDXambKxaWoKbIYQUxjQ8PsAOv12b53t2OH9Tmtw7izQ8xNfseYBi73bfpAWBZhtF4mdfxcYNLakra0diaYHe6nPNNCYFU4xErW47DbnePmRLFhDs%3D) incubated in MSM medium with different Sb(III) concentrations (0, 0.1, 0.3, 0.5, and 1.0 mM) for 24 h at 30°C. Error bars correspond to standard deviations of triplicate means.

**
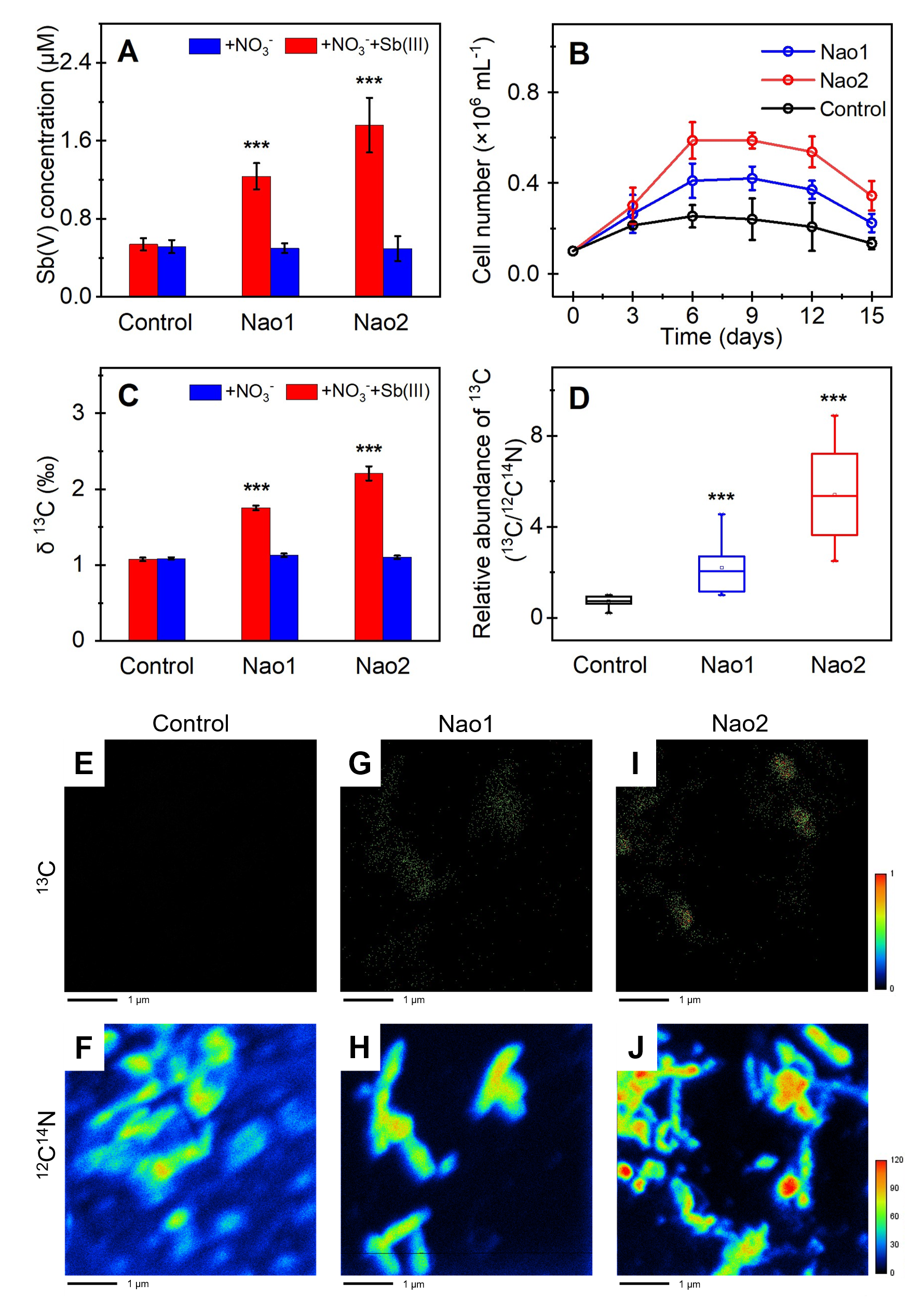
**

**Figure S10. Autotrophic oxidation of Sb(III) in recombinant *R.*** [***palustris***](https://www.so.com/link?m=bI9LsD8hToVzF%2FGxejIC8zIvvtJYcISPhieA3a2vZTY06yT9pBPMph5mr6Mb9IYjHTZ8X2hCZrwwXuNdt0Q9OFhfYJo%2F8kFWzuAqMZWp%2FbErOH5HD11flEdU5hCFMapc8VmqejdRLRO4Qs2j%2FKP60qoUfjDzDXambKxaWoKbIYQUxjQ8PsAOv12b53t2OH9Tmtw7izQ8xNfseYBi73bfpAWBZhtF4mdfxcYNLakra0diaYHe6nPNNCYFU4xErW47DbnePmRLFhDs%3D) **during 15 days.** The amount of Sb(III) oxidation (**A**), cell number monitored over time (**B**), ^13^C incorporation by the [isotope ratio mass spectrometry](https://xueshu.baidu.com/usercenter/paper/show?paperid=b2cd5df6c341a816bd3250d01e85b609&site=xueshu_se) analysis (**C**), relative abundance of ^13^C by nano-SIMS analysis (**D**), nano-SIMS images of ^13^C (**E, G, and I**), and ^12^C^14^N (as an indicator of biomass) (**F, H, and J**) in *R.* [*palustris*](https://www.so.com/link?m=bI9LsD8hToVzF%2FGxejIC8zIvvtJYcISPhieA3a2vZTY06yT9pBPMph5mr6Mb9IYjHTZ8X2hCZrwwXuNdt0Q9OFhfYJo%2F8kFWzuAqMZWp%2FbErOH5HD11flEdU5hCFMapc8VmqejdRLRO4Qs2j%2FKP60qoUfjDzDXambKxaWoKbIYQUxjQ8PsAOv12b53t2OH9Tmtw7izQ8xNfseYBi73bfpAWBZhtF4mdfxcYNLakra0diaYHe6nPNNCYFU4xErW47DbnePmRLFhDs%3D)*-*pCE2TA (Control), *R.* [*palustris*](https://www.so.com/link?m=bI9LsD8hToVzF%2FGxejIC8zIvvtJYcISPhieA3a2vZTY06yT9pBPMph5mr6Mb9IYjHTZ8X2hCZrwwXuNdt0Q9OFhfYJo%2F8kFWzuAqMZWp%2FbErOH5HD11flEdU5hCFMapc8VmqejdRLRO4Qs2j%2FKP60qoUfjDzDXambKxaWoKbIYQUxjQ8PsAOv12b53t2OH9Tmtw7izQ8xNfseYBi73bfpAWBZhtF4mdfxcYNLakra0diaYHe6nPNNCYFU4xErW47DbnePmRLFhDs%3D)-pCE2TA*-nao1* (Nao1), and *R.* [*palustris*](https://www.so.com/link?m=bI9LsD8hToVzF%2FGxejIC8zIvvtJYcISPhieA3a2vZTY06yT9pBPMph5mr6Mb9IYjHTZ8X2hCZrwwXuNdt0Q9OFhfYJo%2F8kFWzuAqMZWp%2FbErOH5HD11flEdU5hCFMapc8VmqejdRLRO4Qs2j%2FKP60qoUfjDzDXambKxaWoKbIYQUxjQ8PsAOv12b53t2OH9Tmtw7izQ8xNfseYBi73bfpAWBZhtF4mdfxcYNLakra0diaYHe6nPNNCYFU4xErW47DbnePmRLFhDs%3D)-pCE2TA-*nao2* (Nao2) during the nitrate-dependent Sb(III) oxidation process. Error bars correspond to standard deviations of triplicate means. Scale bars, 1 μm. Colored scale bars indicate ^13^C or ^12^C^14^N atom percent. Asterisks indicate that Nao1 and Nao2 are significantly different from Control (****P* < 0.001).

**Supplementary Tables**

**Table S1.** The major components of the tested soils.

| **Metal** | **Mean±S.D.**  **(n=3, mmol/g dry weight)** |
| --- | --- |
| Sb | 0.07±0.44 |
| Fe | 0.86±0.33 |
| Ca | 0.35±0.21 |
| Si | 0.09±0.24 |
| Mn | 0.01±0.30 |
| Mg | 0.06±0.40 |
| TOC | 0.17±0.35 |

**Table S2.** Primers used in this study.

| **Name** | **Sequence (5'-3')** | **Location/Target** |
| --- | --- | --- |
| nao1-F | CCCACCCTCCTGTTAAGCGAGTCAGGGCAGATTG | For cloning *nao1* to pCE2TA/Blunt-Zero |
| nao1-R | CGTCTACAGCGGCTCGTCGATGACCAACGAGA | For cloning *nao1* to pCE2TA/Blunt-Zero |
| nao2-F | GAGGAGATCCTGACATCGGACACCCATGGGGC | For cloning *nao*2 to pCE2TA/Blunt-Zero |
| nao2-R | TCTACGGCCCCGGCGATGACCGGCTCCTGCT | For cloning *nao2* to pCE2TA/Blunt-Zero |
| nar-F | CGCTCTAGAACTAGTGGATCCTCTGGCTCTACCTGCTTGAGGA | For cloning *nar* to pBBR1 |
| nar-R | AGGGAACAAAAGCTGGGTACCGCCGCACCGAGCAGCAGC | For cloning *nar* to pBBR1 |

**Table S3**. Thermodynamic constants in the chemical equation of nitrate-dependent Sb(III) oxidation.

| **Substance** | ***G*_f_^o^ (kJ/mol)** | **Ref** |
| --- | --- | --- |
| Sb(OH)_3_ | -513 | [7] |
| Sb(OH)_6_^-^ | -1208 | [7] |
| NO_3_^-^ | -115 | [8] |
| NO_2_^-^ | -29 | [8] |
| H_2_O | -237 | [7] |
| Δ*G*° (kJ/mol) | -135 | This study |
| Δ*G* (kJ/mol)= Δ*G*°+RTlnQ [9] | -147 | This study |

**Table S4.** Assembly statistics for the 9 draft genomes assembled from shotgun metagenomics sequencing library of the treatment amended with Sb(III) and NO_3_^-^.

| **Genome bin I. D.** | **Assigned bin “species” name^a^** | **No. of contigs** | **Size**  **(bp)** | **N50^b^**  **(bp)** | **G + C content (%)** | **Completeness**  **(%)^c^** | **Contamination**  **(%)** |
| --- | --- | --- | --- | --- | --- | --- | --- |
| Bin21 | *Azospirillum* sp. | 166 | 7373155 | 100959 | 69.98 | 98.41 | 3.41 |
| Bin11 | *Microvirga* sp. | 125 | 5354839 | 87742 | 64.17 | 99.37 | 1.53 |
| Bin18 | *Bryobacteraceae* sp. | 59 | 5459431 | 167699 | 63.20 | 99.13 | 0.87 |
| Bin16 | *Symbiobacteriaceae* sp. | 61 | 6367923 | 177214 | 64.46 | 99.01 | 1.68 |
| Bin3 | *Pseudomonas* sp. | 235 | 5976197 | 54243 | 65.31 | 98.86 | 3.72 |
| Bin24 | *Pseudoxanthomonas* sp. | 93 | 3635197 | 125708 | 67.75 | 98.41 | 3.41 |
| Bin14 | *Allorhizobium* sp. | 973 | 4778459 | 5932 | 60.79 | 83.21 | 7.16 |
| Bin9 | *Pseudorhodoplanes* sp. | 962 | 3903002 | 4711 | 63.76 | 82.77 | 4.91 |
| Bin1 | *Noviherbaspirillum* sp. | 980 | 3935582 | 4541 | 59.15 | 68.62 | 2.59 |

^a^Based on LCA of the BLAST output. ^b^N50: number of the largest contigs that sum up to 50% of the total sum of bases. ^c^Based on CheckM output.

**Table S5.** The catalytic subunits containing Mo-bisPGD cofactor found in *Symbiobacteriaceae* (bin 16).

| **Gene ID** | **Length (bp)** | **Protein name** |
| --- | --- | --- |
| gene0576 | 2016 | molybdopterin oxidoreductase family protein |
| gene0694 | 2040 | molybdopterin oxidoreductase family protein |
| gene0742 | 2289 | xanthine dehydrogenase molybdopterin-binding subunit |
| gene1296 | 2271 | nitrate reductase molybdopterin-binding subunit |
| gene1934 | 2382 | NADH-quinone oxidoreductase subunit NuoG |
| gene2689 | 2289 | molybdopterin oxidoreductase family protein |
| gene2774 | 2361 | Dimethyl sulfoxide reductase DmsA |
| gene2470 | 2433 | formate dehydrogenase alpha subunit |
| gene2810 | 2124 | molybdopterin oxidoreductase family protein |
| gene3296 | 2415 | formate dehydrogenase alpha subunit |
| gene3531 | 2070 | formate dehydrogenase subunit alpha |
| gene3676 | 3105 | nitrate reductase alpha subunit |
| gene4093 | 2580 | dimethyl sulfoxide reductase DmsA |
| gene4522 | 2190 | nitrate reductase alpha subunit |
| gene5022 | 1212 | molybdopterin-dependent oxidoreductase |
| gene5294 | 1755 | NADH-quinone oxidoreductase subunit NuoG |
| gene6092 | 849 | carbon-monoxide dehydrogenase subunit |

**References**

1. Chen S, Zhou Y, Chen Y, Gu J. Fastp: an ultra-fast all-in-one FASTQ preprocessor. Bioinformatics. 2018;34:884-890.

2. Magoc T, Salzberg SL. FLASH: fast length adjustment of short reads to improve genome assemblies. Bioinformatics. 2011;27:2957-2963.

3. Callahan BJ, McMurdie PJ, Rosen MJ, Han AW, Johnson AJA, Holmes SP. DADA2: high resolution sample inference from Illumina amplicon data. Nat Methods. 2016;13:581-583.

4. Bolyen E, Rideout JR, Dillon MR, Bokulich NA, Abnet CC, Ghalith GA, et al. Reproducible, interactive, scalable and extensible microbiome data science using QIIME2. Nat Biotechnol. 2019;37:1091-1091.

5. Schloss PD, Westcott SL, Ryabin T, Hall JR, Hartmann M, Hollister EB, et al. Introducing mothur: open source, platform independent, community supported software for describing and comparing microbial communities. Appl Environ Microbiol. 2009;75:7537-7541.

6. Wang L, Ye L, Yu Y, Jing C. Antimony redox biotransformation in the subsurface: Effect of indigenous Sb(V) respiring microbiota. Environ Sci Technol. 2018;52:1200-1207.

7. Sun X, Kong T, Li F, Haggblom MM, Kolton M, Lan L, et al. *Desulfurivibrio* spp. mediate sulfur-oxidation coupled to Sb(V) reduction, a novel biogeochemical process. ISME J. 2022;16:1547-1556.

8. Chauhan R, Srivastava VC. Activity coefficient of multi-ions and Gibbs free energy calculation during electrochemical nitrate reduction in synthetic and actual wastewater. J Chem Thermodyn. 2022;164:106621.

9. Amend JP, LaRowe DE. Minireview: demystifying microbial reaction energetics. Environmen Microbiol. 2019;21:3539-3547.
